# Field evaluation of semi-automated moisture estimation from geophysics using machine learning

**DOI:** 10.1002/vzj2.20246

**Published:** 2023-03

**Authors:** Neil Terry, Frederick D. Day-Lewis, John W. Lane, Carole D. Johnson, Dale Werkema

**Affiliations:** 1 U.S. Geological Survey, New York Water Science Center, 126 Cooke Hall, University at Buffalo North Campus, Buffalo, New York, USA; 2 Pacific Northwest National Laboratory, Richland, Washington, USA; 3 U.S. Geological Survey, Office of International Programs, Storrs, Connecticut, USA; 4 U.S. Geological Survey, Observing Systems Division, Hydrologic Remote Sensing Branch, Storrs, Connecticut, USA; 5 U.S. Environmental Protection Agency, Office of Research and Development, Center for Public Health and Environmental Assessment, Pacific Ecology Systems Division, Newport, Oregon, USA

## Abstract

Geophysical methods can provide three-dimensional (3D), spatially continuous estimates of soil moisture. However, point-to-point comparisons of geophysical properties to measure soil moisture data are frequently unsatisfactory, resulting in geophysics being used for qualitative purposes only. This is because (1) geophysics requires models that relate geophysical signals to soil moisture, (2) geophysical methods have potential uncertainties resulting from smoothing and artifacts introduced from processing and inversion, and (3) results from multiple geophysical methods are not easily combined within a single soil moisture estimation framework. To investigate these potential limitations, an irrigation experiment was performed wherein soil moisture was monitored through time, and several surface geophysical datasets indirectly sensitive to soil moisture were collected before and after irrigation: ground penetrating radar, electrical resistivity tomography (ERT), and frequency domain electromagnetics (FDEM). Data were exported in both raw and processed form, and then snapped to a common 3D grid to facilitate moisture prediction by standard calibration techniques, multivariate regression, and machine learning. A combination of inverted ERT data, raw FDEM, and inverted FDEM data was most informative for predicting soil moisture using a random regression forest model (one-thousand 60/40 training/test cross-validation folds produced root mean squared errors ranging from 0.025–0.046 cm^3^/cm^3^). This cross-validated model was further supported by a separate evaluation using a test set from a physically separate portion of the study area. Machine learning was conducive to a semi-automated model-selection process that could be used for other sites and datasets to locally improve accuracy.

## 1 | INTRODUCTION

Soil moisture, or volumetric water content (VWC), is an important property in a wide variety of fields including ecology (D. A. Robinson et al., 2008), agriculture (Blanchy et al., 2020), and environmental engineering (Straub & Lynch, 1982). Direct measurement of soil moisture with probes is difficult because sensors must physically intrude into the soil, which locally disturbs natural conditions via compaction from probe insertion (Iwata et al., 2017), or borehole installation. In the latter case, poor borehole sidewall contact may result in preferential flowpaths (Doležal et al., 2012).

Various surface geophysical methods are directly or indirectly sensitive to VWC. Ground penetrating radar (GPR) is strongly sensitive to VWC, as water content typically dominates soil dielectric permittivity, which in turn controls radar wave velocity (e.g., Mangel et al., 2022). Electrical resistivity tomography (ERT) and frequency domain electromagnetic induction (FDEM) are both techniques for mapping two-dimensional/three-dimensional (2D/3D) subsurface bulk electrical conductivity (EC) variations that can be used for moisture estimation if porewater possesses an electrical contrast from the soil (e.g., Shanahan et al., 2015). Nuclear magnetic resonance (NMR) technology is directly sensitive to water content (e.g., Parsekian et al., 2019), although this method is typically limited to 1D surface soundings or use in boreholes. D. A. Robinson et al. (2008) and Garré et al. (2021) provide excellent summaries of state-of-the-art methods for soil moisture monitoring, including geophysical methods.

Petrophysical models define a relationship between geophysically measured parameters (EC) and soil/rock properties (VWC). While EC from surface-based geophysics can qualitatively indicate variable VWC, uncertainties in the VWC-EC petrophysical model can lead to large errors in quantitative moisture predictions even in relatively uniform and clay-free lithologies (e.g., uncertainties >28% of total volumetric water content; Tso et al., 2019). Because of this, moisture estimates from surface-based electrical geophysical methods (e.g., FDEM and ERT) may be limited to a qualitative interpretation of approximate zones of relatively high/low moisture unless an acceptable means for estimating variability in petrophysical properties has been established. For example, Steiner et al. (2022) jointly inverted seismic refraction and ERT data collected at high and low frequencies (i.e., induced polarization) to yield spatially continuous estimates of porosity, cation exchange capacity, and VWC. Garré et al. (2011) performed an ERT-based moisture monitoring and root water uptake experiment on a soil monolith using detailed time-domain reflectometry data to characterize different soil horizons in the monolith to calibrate a Waxman and Smits (1968) petrophysical function. These studies highlight the potential of petrophysical model calibration to yield quantitative soil moisture estimates from geophysics; nevertheless, characterizing petrophysical properties in sufficient detail at field scales remains challenging.

Established petrophysical formulations may be too general to estimate intra-site moisture variations within acceptable error bounds. Given the range of factors causing intra-site petrophysical model variability (such as soil porosity, temperature, surface conductance, and/or pore fluid specific conductance), less rigid, data-driven, local VWC-EC petrophysical calibrations may be appropriate, especially those based on machine learning (Moghadas & Badorreck, 2019; Rentschler et al., 2020). Such machine learning (ML) methods may also provide a convenient way to integrate different geophysical data types and/or obviate the need for certain subjective choices made in processing and inversion steps (Terry et al., 2019).

Given the potential of ML methods to improve moisture estimates from geophysics, our objectives in this research were to (1) develop a field-based, multi-parameter geophysical-VWC dataset, (2) evaluate conventional and ML methods for site-scale model development, and (3) rigorously test the performance of the developed models.

## 2 | MATERIALS AND METHODS

### 2.1 | Field site and experiment

**FIGURE 1:**
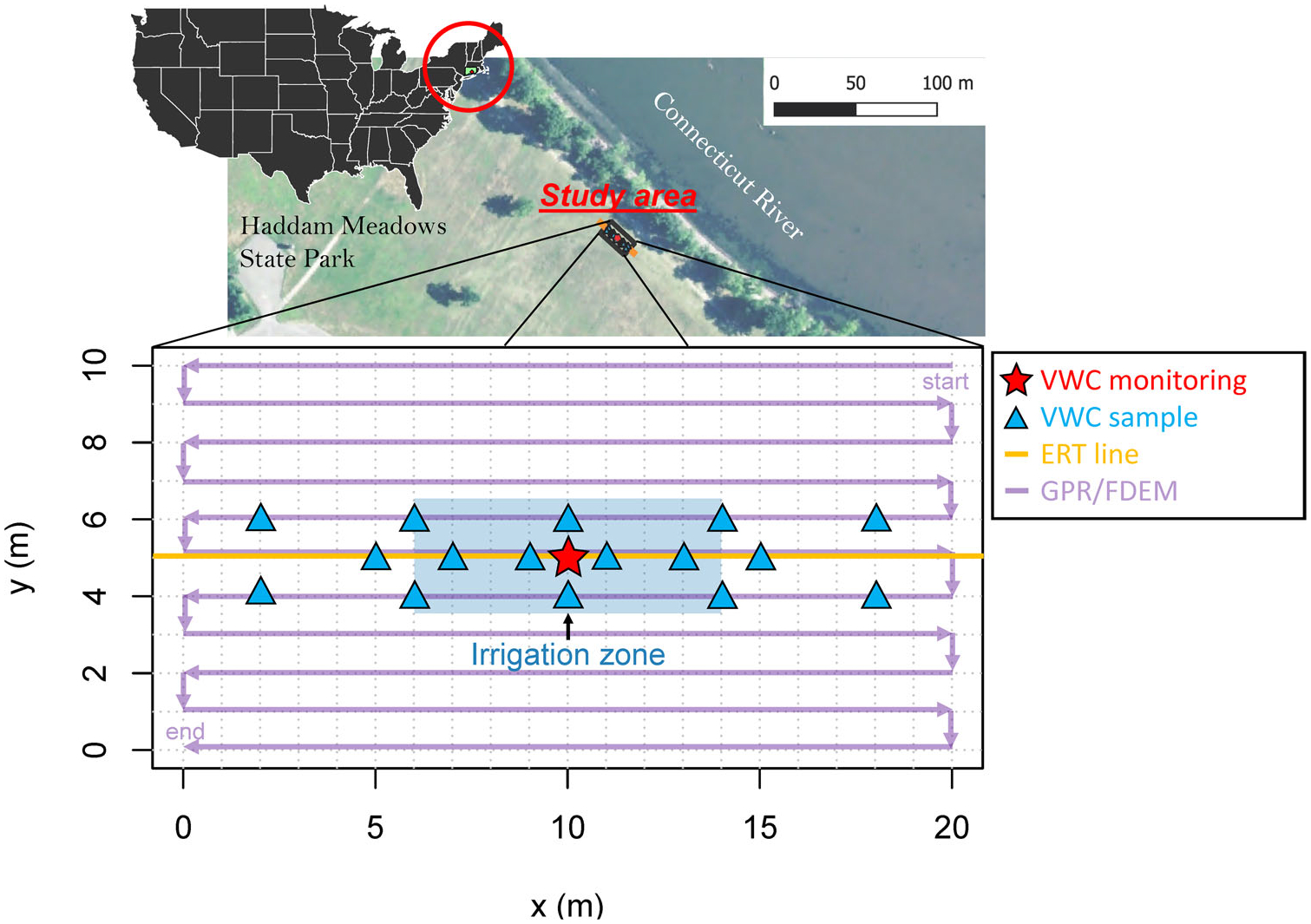
Location of irrigation study area and schematic of experimental setup, Haddam Meadows, Connecticut. Abbreviations: VWC, volumetric water content (soil moisture); ERT, electrical resistivity tomography; GPR/FDEM, ground penetrating radar/frequency domain electromagnetics. Base map image from the National Agriculture Imagery Program

Haddam Meadows, located in the town of Haddam, Connecticut (Figure 1), was chosen as a test site due to its (1) open, mowed field conducive to performing geophysical measurements; (2) proximity to the Connecticut River to supply water for the irrigation experiment; and (3) having relatively simple, well-characterized soils (Stone et al., 2005). Quaternary deposits at the site are about 5 m thick and overly bedrock; the water table is about 2 m below land surface. The Quaternary deposits consist of well-sorted, fine- to medium-grained alluvial sand. Below the sand, a variably thick layer of glaciofluvial coarse sand and gravel overlies glaciolacustrine clays. The focused study area (centered at: 41.485314°, −72.514604°) consisted of a 20− × 10-m plot adjacent to the Connecticut River (Figure 1).

An irrigation experiment was performed at this site on July 16, 2019. Irrigation was conducted over an approximate 8-h period from 3:15 p.m. to 11:15 p.m. Eastern Daylight Time by spraying water over the center of the study grid (an 8− × 3-m area) (Figure 1). Irrigation water was pumped from the Connecticut River into large tubs, premixed with iodized table salt, and stirred until the mixture reached a specific conductance of 1000 μS/cm. Pre-modeling of ERT and FDEM (Terry et al., 2017; see Supporting Information) indicated that natural river water (specific conductance of approximately 100 μS/cm) was unlikely to be detected. Artificially elevating the electrical conductivity of the irrigation water rendered a potentially discernable electrical conductivity signature associated with soil moisture changes during/after the experiment while not harming vegetation. Over the 8-h irrigation period, about 5300 L (1400 gallons) of irrigation water was sprayed, generally evenly, over the 8- × 3-m irrigation area, representing about 221 mm of irrigation. Some of this water was likely lost to evapotranspiration given the high temperature during daylight hours of 35.5°C and the grassy vegetation. Satellite-derived daily evapotranspiration (Senay & Kagone, 2019) from the field experiment location and date was 6.3 mm/day.

Geophysical data collection before and after irrigation included (1) multifrequency FDEM using a Geophex GEM-2 instrument, (2) GPR using an ImpulseRadar Crossover system, and (3) ERT using an AGI Supersting R8 instrument. FDEM data were collected at five frequencies over a broad frequency band (3930–93,090 Hz) in both horizontal coplanar (HCP; ski “flat” parallel to the ground; typical measurement) and vertical coplanar (VCP; ski rotated 90 degrees on side) modes. FDEM data were initially checked with a ferrite rod to ensure the instrument was properly calibrated (producing constant in-phase and zero quadrature for all frequencies), and prior to each FDEM collection event over the study grid, stationary data were collected at a reference location just outside the study area for a period of approximately 1 min to assess any instrumental drift between surveys. GPR data were collected with 170 MHz antennas (CO1760) in common offset configuration triggered by an odometer wheel (a trace was collected every 0.0489 m). Both FDEM and GPR datasets were gathered over the 10− × 20-m study area in 1-m spaced parallel lines in the pattern shown in Figure 1. An ERT line was set up that bisected the center of the grid using 56 stainless-steel electrodes at 0.5 m spacing (Figure 1). ERT data were collected using both dipole–dipole (762 measurements, max *n* = 8, max *a* = 6) and Wenner (418 measurements, max *a* = 5) arrays. For each ERT measurement, two stacks (replicate measurements) were collected. Measurement time was set to 1.2 s.

**FIGURE 2:**
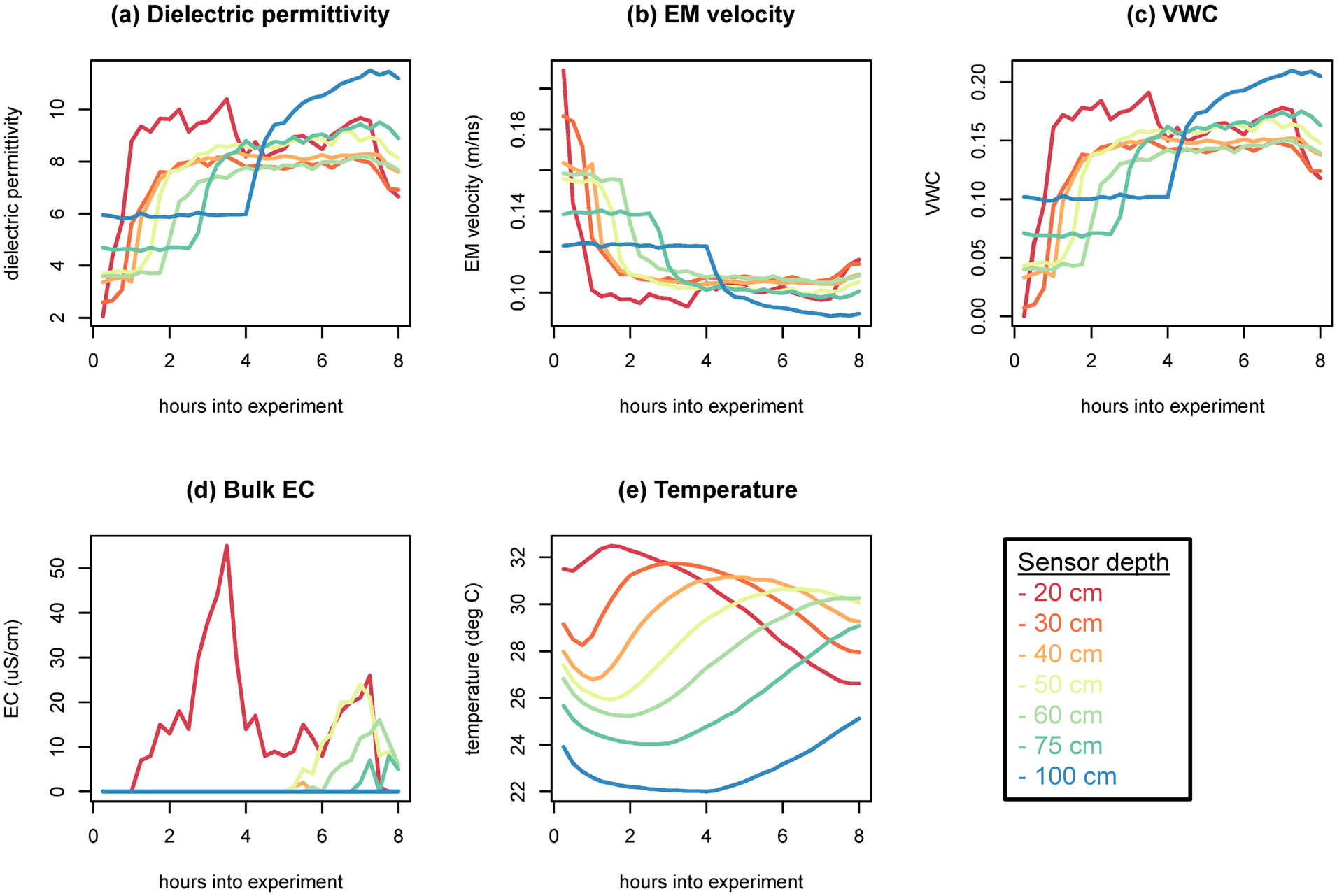
Moisture sensor parameters measured at different depths throughout the irrigation experiment at the center of the study area (red star in Figure 1): (a) dielectric permittivity, (b) electromagnetic (EM) velocity, (c) volumetric water content (soil moisture) (VWC), (d) bulk electrical conductivity (EC), (e) temperature

A Campbell SoilVUE10 time domain reflectometer (TDR) soil moisture and temperature profiler equipped with a battery was installed into a 0.05-m diameter hand-augered hole at the center of the site to record and monitor temperature, dielectric permittivity, and EC at nine depths (5, 10, 20, 30, 40, 50, 60, 75, and 100 cm) during irrigation. The 5-cm and 10-cm sensors were deemed unreliable as the soil surrounding these sensors had been disturbed and partially washed away during probe installation and initial watering. Derived parameters automatically output from this instrument include specific conductance, electromagnetic wave velocity, and VWC. Initial (dry) conditions from this sensor showed slightly increasing VWC with depth (completely dry to 0.1 VWC at 1.0-m depth), decreasing temperature (32–24°C), and EC below the sensitivity of the instrument. Data were recorded at 15-min intervals. VWC showed expected increases with depth through time (Figure 2c), with the deepest sensor (1.0 m) reaching saturation at approximately 7 h into the experiment. Low electrical conductivity values (<20 μS/cm) were measured (Figure 2d) and for deeper sensors arrived later than the observed VWC pulse (Figure 2c), potentially as a natural result of the expected delay between the arrival of the infiltration front and solute front in partially saturated soils, or perhaps from loose sensor sidewall contact initially (i.e., insufficient electrical contact between the SoilVUE EC sensors and the surrounding soil). Temperature showed diurnal temperature variation with the expected dampening and phase shift of the diurnal signal with depth (Figure 2e).

Immediately following irrigation, FDEM, ERT, and GPR datasets were collected in the same manner as done prior to irrigation and as shown in the pattern in Figure 1. Additionally, several point-based moisture and temperature measurements were gathered with an Aquaterr EC-350 push probe at discrete depths (ranging from just below the surface to 0.75-m depth). This analog instrument reports moisture as a percentage of VWC as an onscreen readout and therefore requires an estimate of porosity for conversion to VWC. Porosity was assumed to be 0.21 based on previous neutron logging performed at nearby wells as this site (Buursink et al., 2002). In total, 16 unique locations were sampled (Figure 1) at various depths to provide 59 point-based moisture estimates. The last temporal dataset from the SoilVUE sensor was combined with these values to produce a VWC dataset of 66 moisture values for model training and testing purposes.

**FIGURE 3:**
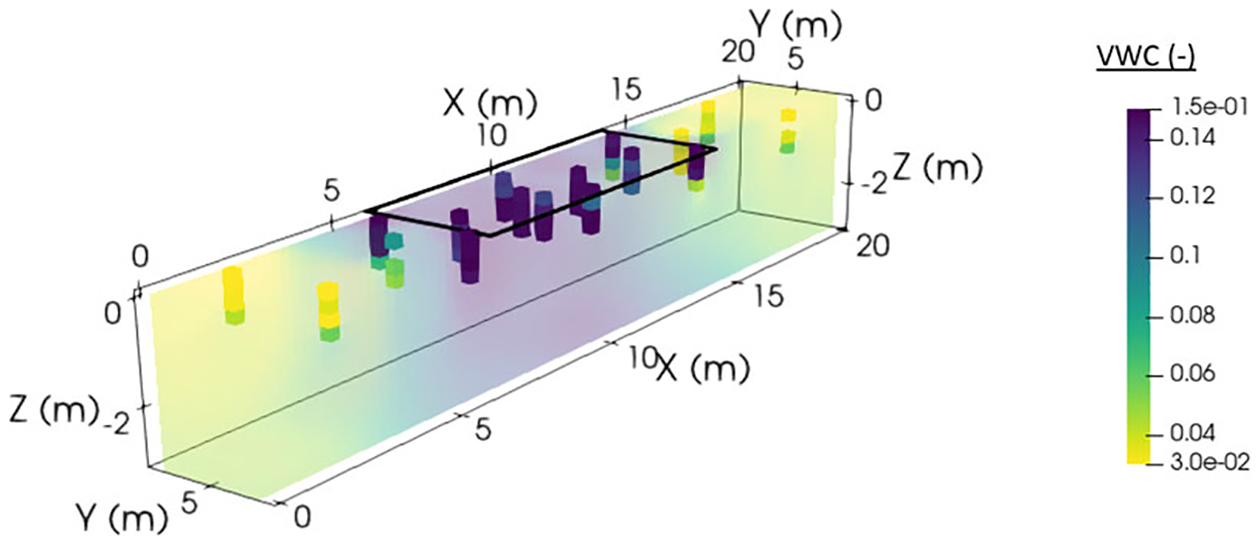
Measured moisture values (volumetric water content) post-irrigation (solid blocks) and inverse-distance-weighted interpolation of those values (weighting parameter = 2, shown as semi-transparent)

Our data analysis focused on this post-irrigation VWC dataset, as (1) we did not want to disrupt the soil prior to/during irrigation with invasive push probe measurements and (2) post-irrigation showed the most substantial contrasts in terms of soil moisture and geophysical data. These measured post-irrigation moisture values are shown in Figure 3, with the center of the irrigated zone showing close to saturated VWC values (0.21), edge areas at depth showing intermediate values, and outside-of-irrigation areas showing low values of 0.03 or less, sometimes slightly increasing with depth.

### 2.2 | Geophysical data preparation

All geophysical data required underwent a level of basic processing before being used. First, each dataset was georeferenced. ERT electrode positions were gathered using a total station. FDEM on-board GPS accuracy was not suitably accurate for the small area and fine resolution desired for this study, so line start and end points were also collected with a total station. FDEM data positions were then corrected to these known points by assuming a constant walking speed along individual lines and linearly interpolating positions. Although individual lines were not demarcated in the GPR data files, a differential fix was maintained with the on-board GPS, so positional accuracy was relatively accurate to at least the decimeter scale (ascertained by plotting the raw data positions and observing minimal drift and no crossover between parallel lines). Regions of data outside the study area (i.e., where the GPR was turned around to begin a new line) were manually trimmed out by examining GPS positions and the line start/end points from the total station data. After georeferencing, all data were rotated and translated to the 20− × 10-m local coordinate system (Figure 1) used in this study.

### 2.3 | GPR data

**FIGURE 4:**
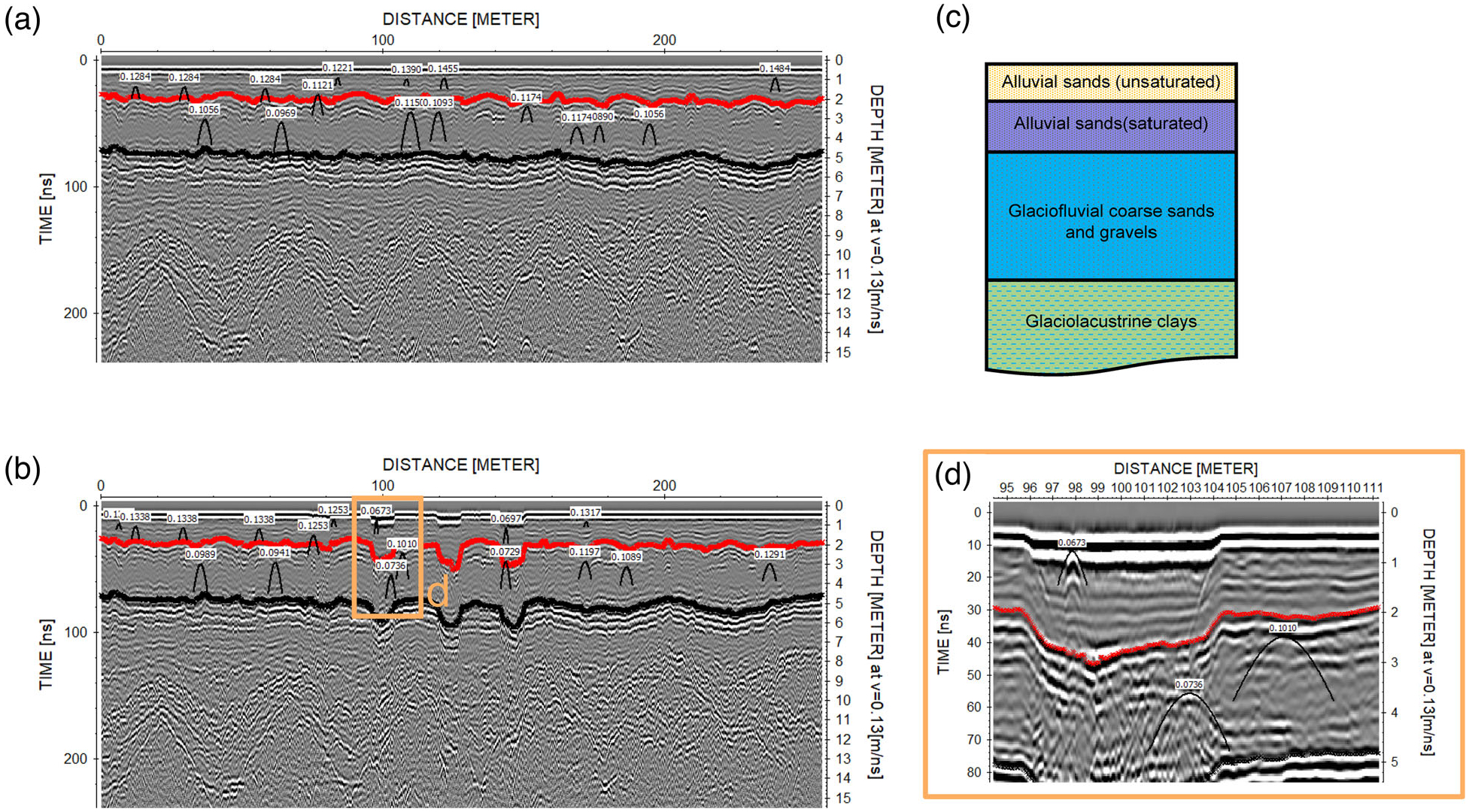
Example processing of Haddam Meadows, Connecticut ground penetrating radar (GPR) data showing layer picks and diffraction hyperbolae used for velocity estimation. (a) Pre-irrigation data, (b) post-irrigation data, (c) known Quaternary layers observed at field test site, (d) zoom on irrigated area showing diffraction hyperbola picks. The red lines in (a), (b), and (d) are the picked water table reflector, and the black lines are the picked glaciofluvial sands reflector.

Raw GPR data were corrected for time zero. Pre- and post-irrigation GPR data were further processed in ReflexW (version 9.5.1, Sandmeier Geophysical Research). Data were imported using the “IMPULSERADAR” setting, and dewow and AGC-gain were applied to enhance the contrasts of reflectors. The processed radargrams are shown in Figure 4. Pre-irrigation GPR data (Figure 4a) clearly show the water table and known layers in the Quaternary deposits (alluvial sand over glaciofluival sand and gravel over glaciolacuvstrine clays, Figure 4c). The effect of irrigation is also clearly visible in post-irrigation GPR data as later two-way travel times to the first and second layer reflectors (Figure 4b), resulting from increases in soil moisture slowing GPR wave velocities in these areas.

**FIGURE 5:**
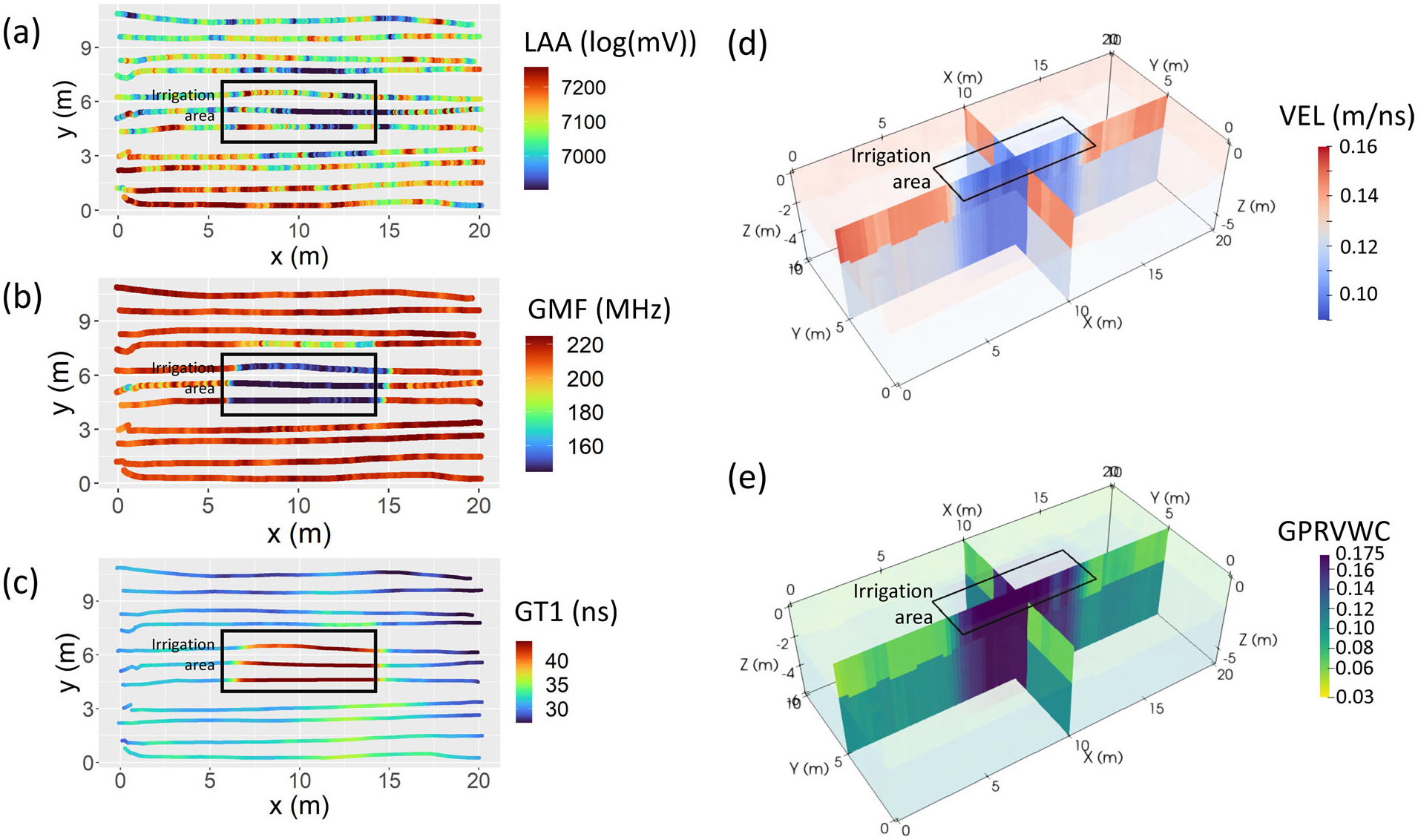
Ground penetrating radar (GPR)-derived datasets for the irrigation experiment in Haddam Meadows, Connecticut: (a) summed log absolute amplitudes (LAA), (b) trace GPR mean frequencies (GMF), (c) two-way travel time to the water table reflector (GT1), (d) electromagnetic wave velocity (VEL), (e) GPR-derived volumetric water content using Topp’s equation (GPRVWC)

For pre- and post-irrigation datasets, two-way arrival times to the water table and alluvial-glaciofluvial reflector were picked as shown in Figure 4a,b. Diffraction hyperbolae were fit with velocities within each of these layers in the pre-irrigation dataset to establish root-mean-square velocities for the vadose zone and the saturated zone (Figure 4a,b,d). Pre-irrigation root-mean-square velocities for the two layers were 0.13 ± 0.01 (9 hyperbolae) and 0.11 ± 0.01 (7 hyperbolae) m/ns, respectively. Pre-irrigation spatially extensive depth to water table was computed using the post-irrigation vadose zone velocity, which was estimated assuming that the water-table depth did not change and that all apparent changes to two-way arrival times from this layer were solely due to changes in VWC. From this information, we were able to construct a 3D GPR velocity field post irrigation, to form the VEL (GPR velocity) dataset (Figure 5d). These GPR EM velocity values (Figure 4d) were reasonably consistent with those from the SoilVue sensor (Figure 2b).

Velocity, *v*, was converted to dielectric permittivity (*ε*_*r*_) of the soil as,

(1) $$\varepsilon_{r}={\left(\frac{c}{v}\right)}^{2}$$

where *c* is the speed of light in a vacuum (= 0.3 m/ns). Finally, *ε*_*r*_ was converted to VWC through Topp’s empirical equation (Topp et al., 1980),

(2) $$\text{VWC}=-5.3\times 10^{-2}+2.92\times 10^{-2}\varepsilon_{r}-5.5\times 10^{-4}\varepsilon_{r}^{2}+4.3\times 10^{-6}\varepsilon_{r}^{6}$$

These GPR-estimated VWC values formed the GPRVWC dataset (Figure 5e).

For this study, we were interested in evaluating the performance of explanatory variables derived from raw or minimally processed geophysical data. To this end, we extracted three variables from the post-irrigation GPR dataset:

1. the summed log absolute amplitude (LAA), which were computed by taking the logarithm of the measured absolute voltages along each trace and summed (Figure 5a);
2. the GPR mean frequency (GMF), computed as the mean of the fast-Fourier-transformed data for each GPR trace (Figure 5b);
3. the picked two-way travel time to the water table reflector (GT1, Figure 5c).

### 2.4 | FDEM and ERT data

Pre-irrigation FDEM data were dominated by noise and possessed no discernable spatial patterning in any frequency (data not shown), which is consistent with the overall highly electrically resistive environment conducive to good GPR imaging. Similarly, pre-irrigation ERT data and inversion results showed a lack of discernable patterning and overall low electrical conductivity (<20 μS/cm, data not shown). Electrodes possessed high contact resistance (at times, ~5 kΩ despite efforts to reduce contact resistance), which apparently affected raw data and inversion results and resulted in an inability to image lithology.

Post-irrigation FDEM data were corrected for instrumental drift. Corrections were applied to the post-irrigation dataset based on the difference in mean values (relative to pre-irrigation data) recorded for each frequency. For HCP quadrature data, the corrections applied were −24.08, −36.45, −52.92, +6.148, and +225.9 ppm for the 3930, 8670, 19,110, 42,210, and 93,090 Hz frequencies, respectively. For the VCP quadrature data, the corrections applied were −55.66, −64.22, −62.71, +20.95, and +251.8 ppm.

**FIGURE 6:**
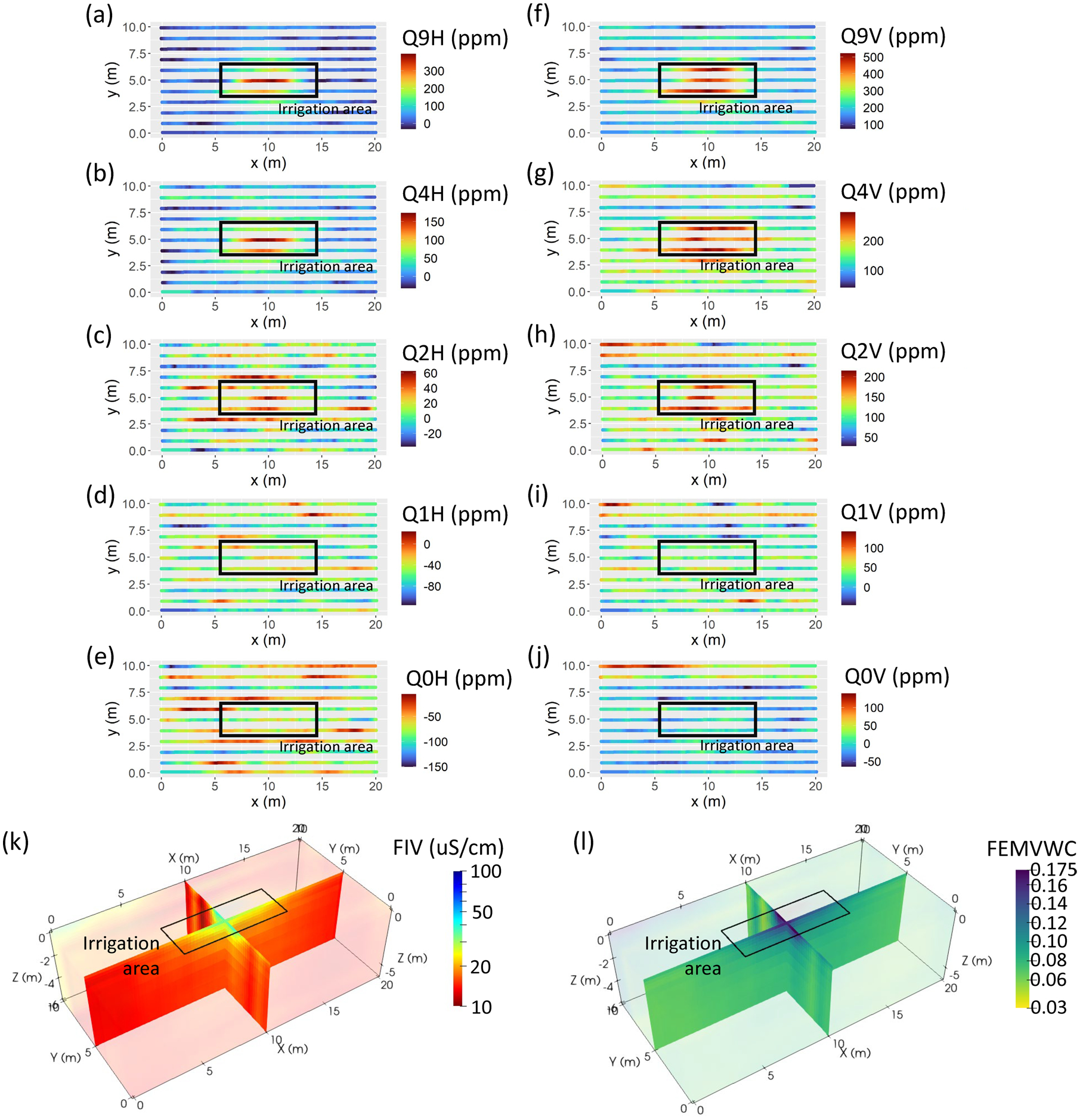
Frequency domain electromagnetics (FDEM)-derived datasets from the irrigation study in Haddam Meadows, Connecticut: (a) horizontal coplanar data collected at 93,090 Hz (Q9H), (b) horizontal coplanar data collected at 42,210 Hz (Q4H), (c) horizontal coplanar data collected at 19,110 Hz (Q2H), (d) horizontal coplanar data collected at 8670 Hz (Q1H), (e) horizontal coplanar data collected at 3930 Hz (Q0H), (f) vertical coplanar data collected at 93,090 Hz (Q9V), (g) vertical coplanar data collected at 42,210 Hz (Q4V), (h) vertical coplanar data collected at 19,110 Hz (Q2V), (i) vertical coplanar data collected at 8670 Hz (Q1V), (j) vertical coplanar data collected at 3930 Hz (Q0V), (k) inverted, temperature-corrected FDEM electrical conductivity (FIV), (l) FDEM-derived volumetric water content using Archie’s Law (FEMVWC)

All raw FDEM data were then spatially smoothed with a 1-m moving averaging window. HCP and VCP quadrature data were matched to common X-Y positions in a data table, such that for each spatial point in the file there were a total of 10 data points available (HCP and VCP coil orientations quadrature each at five measured frequencies). These 10 datapoints comprised quadrature data in HCP orientation (Q0H, Q1H, Q2H, Q4H, and Q9H) and quadrature data in VCP orientation (3930 Hz = Q0V, 8670 Hz = Q1V, 19,110 Hz = Q2V, 42,210 Hz = Q4V, and 93,090 Hz = Q9V). In the following sections, we reference collective groups of raw post-irrigation FDEM information as qHCP (Figure 6a–e) and qVCP (Figure 6f–j), respectively. The qHCP and qVCP data show relative increases when crossing the irrigated area in the upper three frequencies (Figure 6a–c,f–h) but little visible change in the lower frequencies (Figure 6d,e,i–j).

FDEM data were inverted in Aarhus Workbench (v. 6.6.0.2, Aarhus GeoSoftware) using the ground-conductivity meter (GCM) module. These data described above were imported into the software, assuming a 2% relative error and 50 ppm absolute error. A smooth, laterally constrained inversion was performed using quadrature data from all five frequencies. Electrical conductivity was estimated at 20 log-spaced layers from the ground surface to a 10-m depth at each spatial location. Depth of investigation (DOI) calculations that estimate the approximate reliable depth of sensitivity of results were calculated within the software during the inversion. The DOI averaged around 3 m. The acronym given to electrical conductivity output from the FDEM inversion was FIV (Figure 6k).

ERT data were automatically filtered based on either negative or very high (>10,000 Ωm) apparent resistivity, or stacking errors greater than 1%. This filtering removed 28% of the dipole–dipole data and 19% of the Wenner data, mostly attributed to data being above the high apparent resistivity cut-off and likely due to dry conditions outside the irrigation area and associated high contact resistances.

**FIGURE 7:**
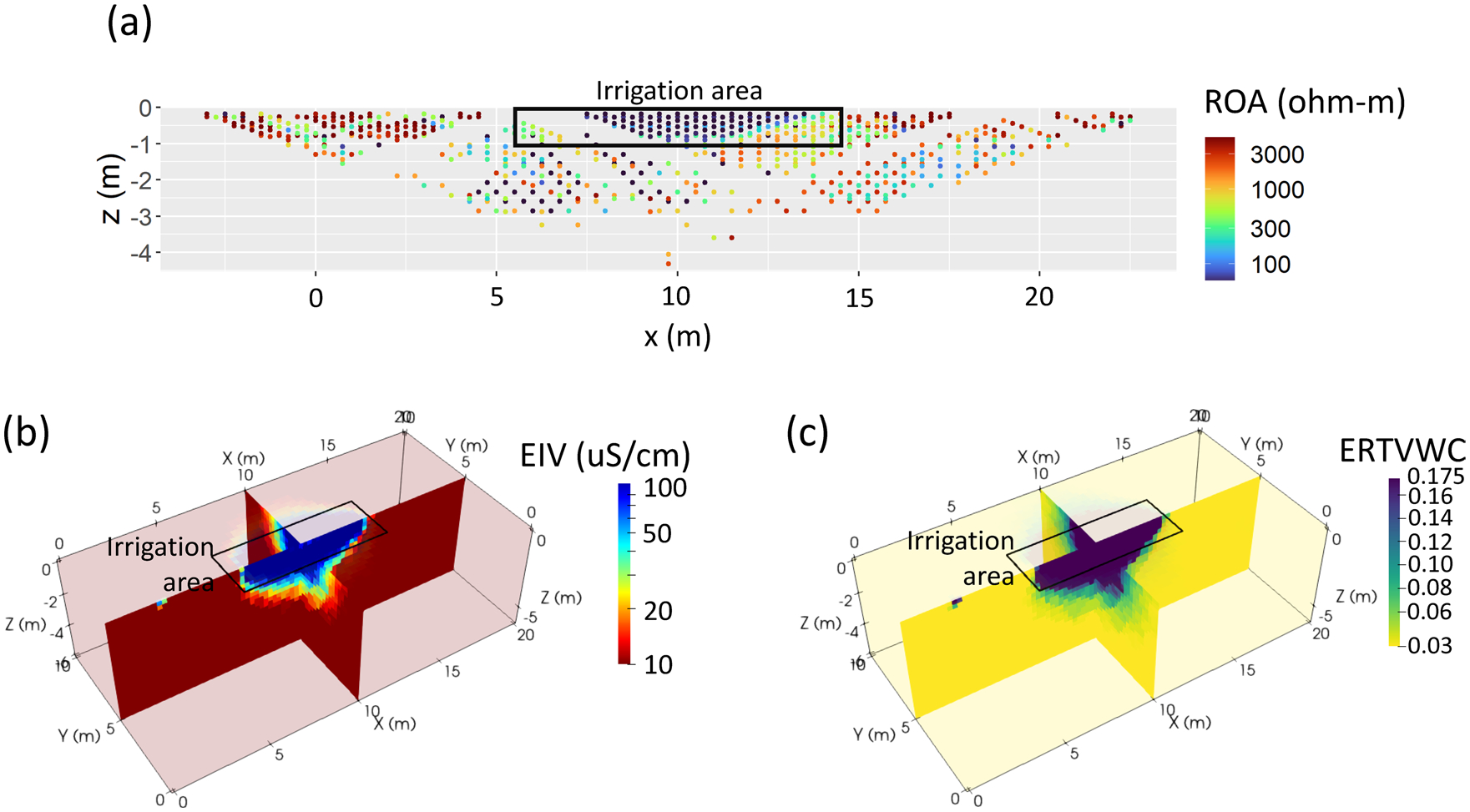
Electrical resistivity tomography (ERT)-derived datasets: (a) pseudosection of raw apparent resistivity (ROA), (b) inverted, temperature-corrected ERT electrical conductivity (FIV), (c) ERT-derived volumetric water content using Archie’s Law (ERTVWC)

Apparent resistivity datapoints were positioned on pseudo-sections using conventional means (*X*-*Y* position at the center point of the current and potential dipole pairs), depth of 52% of the interelectrode spacing for Wenner array data (Loke, 2001), and 18% of the inter-dipole spacing for dipole–dipole array data (Edwards, 1977). The post-irrigation Wenner and dipole–dipole pseudosection data were combined and formed the ROA (raw apparent resistivity) dataset (Figure 7a).

ERT data were inverted in R3t (v. 4.02, Andrew Binley, Lancaster University): a freely available Windows application for performing Occam’s 3D ERT inversions (Binley & Kemna, 2005). ERT inversions fit an earth resistivity model to data subject to spatial smoothness constraints and estimated data errors. A fully 3D mesh was constructed using GMSH (Geuzaine & Remacle, 2009) with characteristic length of mesh elements at the electrodes set to 0.1 m. Element sizes gradually increased away from the electrodes to the edge of the mesh domain (300 m in all directions) to a maximum characteristic length of 50 m. For the inversion, 3D current flow was modeled using this 3D mesh.

To determine convergence, a variable error model was used, with high errors (error intercept = 10 Ω, error slope = 20%) for data where at least one electrode was outside of the irrigation area and lower errors (error intercept = 0.01 Ω, error slope = 10%) for data where all electrodes existed within the irrigation area. This error model distinction was made due to high contact resistances of several kiloOhms (and lower data quality) outside of the irrigation area versus relatively low contact resistances (<1 kΩ) within the irrigation area. For each of the datasets, the inversion converged in a few iterations. The acronym given to EC output from the post-irrigation ERT inversion was EIV (Figure 7b). Low resistivity/high EC zones (<100 ohm-meters/>100 μS/m) clearly associated with the irrigation area are visible in both the raw ERT pseudosection (Figure 7a) and the inversion result (Figure 7b) against a resistive background of several thousand ohm-meters.

EC inversion results from FDEM and ERT (FIV and EIV) were standardized to depth-variable temperature (*T*_std_ = 25°C) following the method of Hayley et al. (2007),

(3) $${\text{EC}}_{\text{std}}=\text{EC}\left[\frac{k\left(T_{\text{std}}-25\right)+1}{k(T-25)+1}\right]$$

where *T* is the local soil temperature and *k* is a constant, set to 0.0183 as empirically determined for a variety of soil types by Hayley et al. (2007). To provide spatially variable temperature information to perform the correction in Equation (3), a 1D, linearly interpolated temperature model from 0.05 to 1.0 m depth was established using available SoilVUE data. Temperature was assumed to stabilize below 1.0 m depth. Since we lacked deeper temperature data, and there are no training/test VWC data points below this depth, this temperature model was deemed adequate for purposes of this experiment. Effectively, this correction caused a maximum adjustment of approximately 10% to inverted EC values.

Finally, temperature corrected EC data were converted to VWC using Archie’s Law (Archie, 1942) for unsaturated sediments and assuming no surface conduction effects, rearranged for VWC,

(4) $$\text{VWC}=\phi {\left(\frac{{\text{EC}}_{\text{std}}}{{\left(\phi_{\text{int}}\right)}^{m}\sigma_{w}}\right)}^{\frac{1}{n}}$$

where *ϕ*_*int*_ and *ϕ* are interconnected and total porosity, respectively, *σ*_*w*_ is pore water conductivity, *m* is an exponent related to soil structure, and *n* is an exponent relating conductivity and water saturation. Here, we assumed values for clean, unconsolidated sands of *ϕ*_int_ = *ϕ* = 0.21, *m* = 1.3 (Archie, 1942), *n* = 1.3 (Schön, 1996), and *σ*_*w*_ = 1000 μS/cm. These formed the FEMVWC (Figure 6l) and ERTVWC (Figure 7c) datasets, respectively. Although the value for porosity is lower than might be expected, we choose this value as it is consistent with other data collected at the site.

Data prepared were interpolated or extrapolated to a common estimation grid covering the area from *X* = 0 to *X* = 20 m, *Y* = 4 to *Y* = 6 m (Figure 1), and *Z* = −3 to *Z* = 0 m, where *Z* = 0 is the ground surface and larger negative values indicate greater depth. The grid consisted of 81 elements in the *X* direction, nine elements in the Y direction, and 13 elements in the *Z* (depth) direction. Element volumes were 0.25 m^3^. Bilinear interpolation was used to assign within-bounds data values to elements. Raw FDEM (qHCP, qVCP) and GPR (LAA, GMF, GT1) were available in X and Y directions only; thus, the same value was used for each *Z* element corresponding to unique *X*-*Y* pairs on the estimation grid. ERT raw data were also only available in X and Z directions; thus, these values were similarly extruded outward over the limited 2-m *Y* domain. Direct VWC data were snapped to the nearest element in the estimation grid. Most VWC measurements were located within unique grid elements; however, some of the more finely spaced measurements from the SoilVUE sensor had two measurements within a single grid cell. In this latter case, both VWC values occupying a grid cell were used in training models and evaluating model performance. Table 1 gives descriptions of each of the explanatory variables derived from geophysics.

### 2.5 | Moisture estimation and ML models

Data were compiled into a data table as a complete post-irrigation slice-in-time dataset, with 66 direct VWC values (independent variable) and 17 possible explanatory variables each with an assigned numeric value and no missing information. The three geophysically derived moisture estimates (FEMVWC, GPRVWC, and ERTVWC) were not included as possible explanatory variables for model development but were instead reserved to compare with other moisture estimation models.

In the following sections, we briefly outline the moisture estimation approaches evaluated, including the MoisturEC software and three different ML algorithms (multivariate regression, support vector regression, and random regression forests), and the specific implementations we used. Details of the ML algorithms are presented in the Appendix.

#### 2.5.1 | MoisturEC

MoisturEC is R-based software for calibrating and combining EC from geophysical inversions and discrete moisture estimates in a single estimation framework, using optimized Tikhonov regularization to balance data fit with model smoothness in the final VWC estimate (Terry et al., 2018). MoisturEC was tested as a means for combining ERT and FDEM inversion results with VWC data to estimate moisture (to compare to results from the ML approaches evaluated in this study). FIV and EIV data were used as a combined EC input to the MoisturEC. Errors were approximated as 1% for the VWC data, 5% for the EIV data, and 50% for the FIV data. The “use data” option to calibrate EIV and FIV values to VWC was employed.

#### 2.5.2 | Multivariate linear regression

Though a classical approach, multivariate linear regression (MVR) (also known as multiple linear regression) is similar to and sometimes considered an ML method given that it can be applied to large datasets to train models of varying complexity, including covariances and variable transformations. Outliers can strongly influence models, and nonlinear relationships may be problematic. However, the simplicity of MVR is a key benefit compared to other ML models where the mechanics are more difficult to trace and understand (e.g., Nguyen et al., 2021). The built-in R function lm (R Core Team, 2021) was used to evaluate, train, and test MVR models. We did not include interaction terms in the MVR models evaluated.

#### 2.5.3 | Support vector regression

Support vector regression (SVR) (Vapnik, 1995) is an adaptation of the support vector machine classification method to regression problems. SVR is suitable for nonlinearity and is resistant to outliers. This method has been applied to various hydrogeophysical problems such as hydraulic conductivity estimation from soil electrical spectra (Boadu, 2020) and soil moisture estimation from airborne and other datasets (Acharya et al., 2021; Achieng, 2019; Jin et al., 2021; Pasolli et al., 2011).

SVR was performed using the R e1071 package (D. Meyer et al., 2021) function svm. The tunable parameters were set to *C* = 1.0 and *ε* = 0.1, respectively (defaults). The internal scaling of variables performed by the svm function often makes the default choices of *C* and ε reasonable regardless of absolute input data magnitudes. A radial basis function was used (default for svm).

#### 2.5.4 | Random regression forests

Random regression forests (RRF) are a class of ensemble prediction algorithms that use many decision trees to develop models (see the Appendix for details of the approach). Of the three methods presented here, RRFs are at the furthest extreme in terms of highest accuracy at the cost of model complexity. They are known to perform very well for large datasets with outliers and complex nonlinear relations between the independent variable and predictor variables, but can be subject to overfitting, biased predictions, and are not suitable for extrapolating outside the range of training conditions (Hengl et al., 2018). Environmental datasets and particularly geophysical data are often spatially and/or temporally autocorrelated, which can result in biased sampling and cross-validation that may give a false impression of robust RRF performance despite having no transferable value (Terry et al., 2021). This issue can be partially addressed by using spatiotemporal cross-validation strategies, such as “Leave-Location-Out” wherein a spatial group of data are withheld for model testing, as opposed to random training-test splits used in k-fold cross-validation (H. Meyer et al., 2018).

We used the R package randomForest (Liaw & Wiener, 2002) with default parameters, except for *ntree* (the number of regression trees to grow), which we set to 101. Each regression tree used the default *m_try* = *p*/3, where *p* is the number of explanatory variables being evaluated in the model.

### 2.6 | Model tests

An initial “all-in” test was performed, wherein ML models for each method (MVR, SVR, and RRF) were trained on the full data table (i.e., using all explanatory geophysical variables and VWC observations) and used to provide VWC values over the full estimation grid. Next, we performed testing of models developed with almost all combinations of parameters. The total number of tests required to do this is given by summing results from the combination formula, for example,

(5) $$\text{total tests }=\overset{p}{\underset{i}{\sum }}\frac{p!}{i!(p-i)!}$$

**FIGURE 8:**
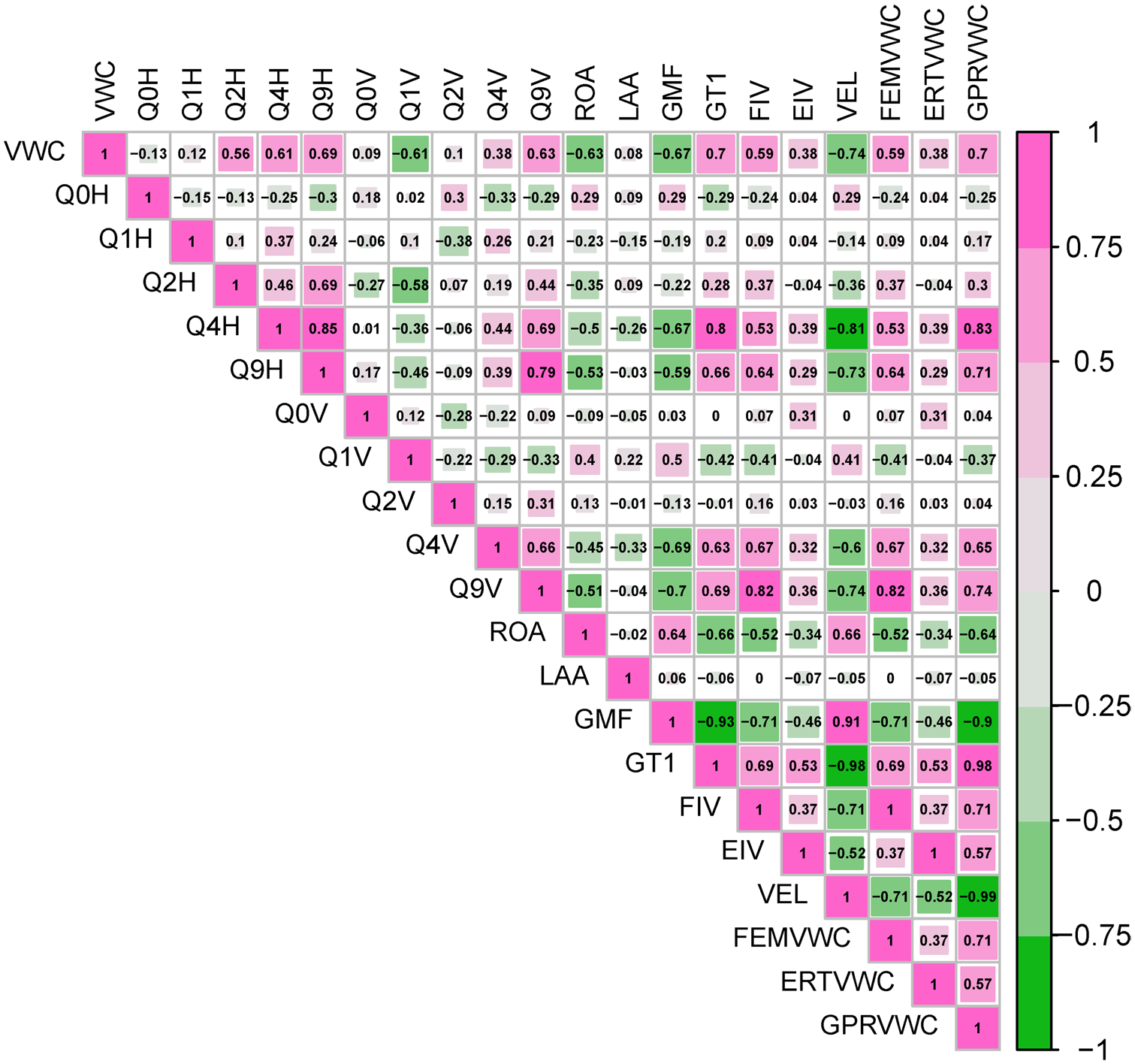
Correlogram (corrgram) of linear correlations between volumetric water content and raw and processed data generated in the irrigation study in Haddam Meadows, Connecticut. See Table 1 for description of acronyms.

Excluding the variables of VWC derived from geophysics using petrophysical models (FEMVWC, GPRVWC, and ERTVWC), this left us with *p* = 17 variables, which would result in 131,071 required tests for each method. To reduce the number of tests, we removed parameters with low linear correlations (<0.3) to VWC (Figure 8), which included Q0H, Q1H, Q0V, Q2V, and LAA. Despite its high (negative) linear correlation to VWC, Q1V was also removed from analysis given the quadrature response from a conductivity meter should be positively correlated with subsurface conductivity. Following this parameter reduction, *p* = 11 variables remained, reducing the number of required tests needed to 2047.

For each of the tests, 1000-fold cross-validation was performed wherein 60% of the data observations were used to train an ML model, and 40% were used to test predictions, as evaluated by the coefficient of variation (*R*^2^) and root-mean-squared error (RMSE) between observed VWC and predicted VWC. The datapoints used in training/test splits were randomly shuffled for each fold of the cross-validations.

All combinations of variable groups (with 1000 cross-validation folds each) were evaluated for each of the ML algorithms described here (MVR, SVM, and RRF). In total, 6,141,000 models were trained and tested.

The relative importance of individual parameter groups to ML models was assessed by the median *R*^2^ value produced from all tests associated with that parameter group. The “optimal” models were chosen based on having the highest median *R*^2^ value across all tests. The parameter groups used to construct these models were then subjected to a more rigorous training/test split, using a physically divided dataset to evaluate the intra-site transferability of the models (i.e., leave-location-out cross-validation). In this final step, less than half of the data (*x* = 0 to *x* = 9.5 m) were used to train models using the identified best set of parameters, and the other portions (*x* = 9.5 to *x* = 20 m) were used to test predictions. This training/test split was then evaluated in reverse (using *x* = 0 to *x* = 10.5 m as the test set). These tests were referred to as “transferability test #1” and “transferability test #2”, respectively.

## 3 | RESULTS

### 3.1 | Moisture model evaluation

**FIGURE 9:**
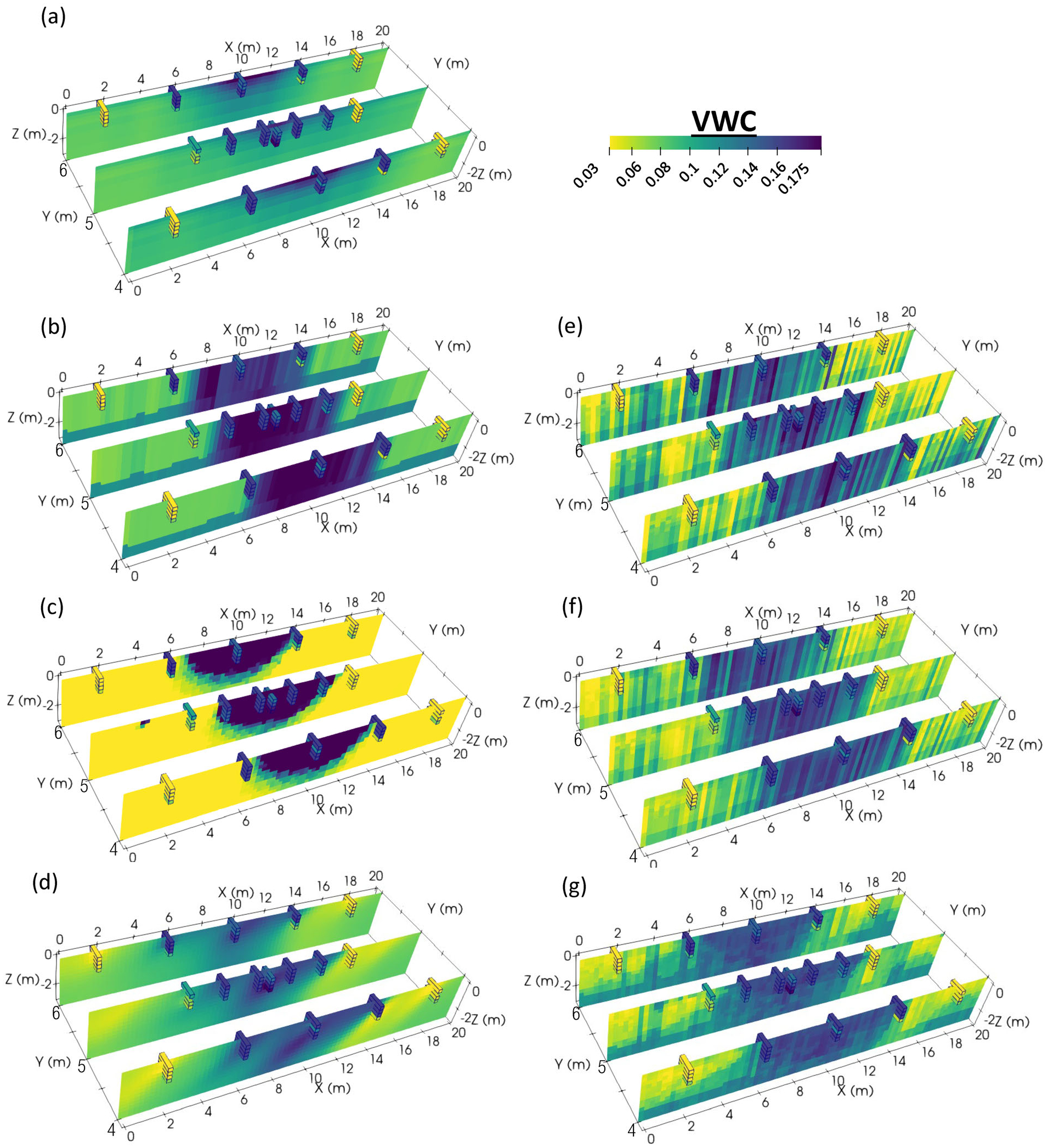
Petrophysical models of volumetric water content (soil moisture) (VWC), R-based moisturEC estimate, and machine learning (ML) models using all data and no cross-validation: (a) frequency domain electromagnetics (FDEM) moisture through Archie’s Law, (b) ground penetrating radar (GPR) moisture through Topp’s equation, (c) electrical resistivity tomography (ERT) moisture through Archie’s Law, (d) MoisturEC result, (e) multivariate regression (MVR) results, (f) support vector regression (SVR) result, (g) random regression forest (RRF) result. Discrete soil moisture points are shown as outlined blocks.

**FIGURE 10:**
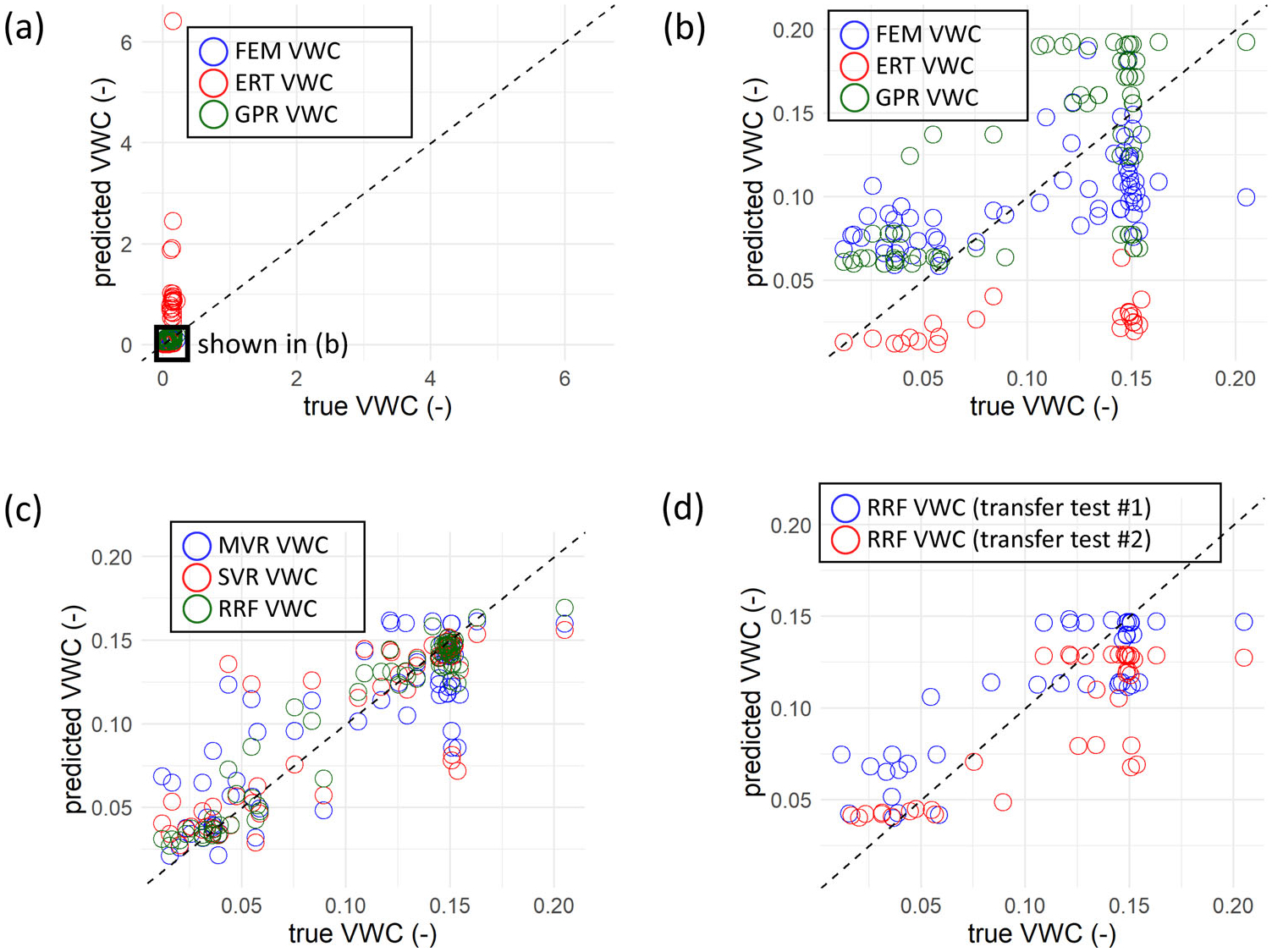
Cross-plots of measured volumetric water content (VWC) versus VWC predictions for irrigation experiment at Haddam Meadows, Connecticut: (a) full range of FEMVWC, ERTVWC, and GPRVWC versus VWC; (b) result in (a) for range of measured VWC values; (c) performance of multivariate regression (MVR), support vector regression (SVR), and random regression forest (RRF) machine learning (ML) methods using all data and explanatory variables; (d) performance of RRF method in the two transferability tests

Results from various moisture predictions are described here and summarized in Table 2. Discrete post-irrigation moisture estimates overlain on petrophysical model-derived VWC are shown in Figure 9a (FEMVWC, moisture from Archie’s Law using the FDEM inversion result), 9b (GPRVWC, moisture from Topp’s equation using interpreted GPR velocities), and 9c (ERTVWC, moisture from Archie’s Law using the ERT inversion result). Cross-plots showing comparisons of measured VWC values versus FEMVWC, ERTVWC, and GPRVWC are shown in Figure 10a,b. The FEMVWC result overpredicts measured VWC in dry areas and underpredicts VWC in irrigated areas (Figure 10b), but generally captures the irrigation area in terms of relative values (Figure 9a) and shows the lowest RMSE (0.043). GPRVWC shows the second most accurate comparison to measured VWC (RMSE = 0.045, Figure 10b); however, the bottom of the irrigated bulb is not captured (Figure 9b). ERTVWC shows the highest errors and is unrealistically overestimated (i.e., greater than the total porosity) at many points (Figure 10a) and underestimated at others (Figure 10b), though the actual volume of the irrigation area is well-defined except for some of the edge measurements (Figure 9c).

VWC values output from MoisturEC using the full dataset are shown in Figure 9d. As this software is designed to honor measured moisture values within the input level of error, the accuracy of moisture predictions from the software was evaluated by leaving out over half of the data for testing as described previously. RMSE values for the transferability test #1 and #2 data points were 0.037 (for the *x* = 9.5 m to *x* = 20 m test set #1) and 0.053 (for the *x* = 0 m to *x* = 10.5 m test set #2), respectively.

ML VWC results from the “all-in” tests (all parameters and all observations used) are shown in Figures 9e (MVR), 9f (SVR) and 9g (RRF). These models all had very high accuracy at the discrete measurement points (*R*^2^/RMSE of MVR =0.71/0.028, SVR = 0.76/0.026, and RRF = 0.94/0.013). However, these all-in models performed relatively poorly when subjected to the transferability tests (Table 3) achieving *R*^2^ values of 0.11–0.37 (MVR), 0.61–0.67 (SVR), and 0.53–0.79 (RRF), underscoring the need for rigorous cross-validation procedures when evaluating the performance of ML models.

**FIGURE 11:**
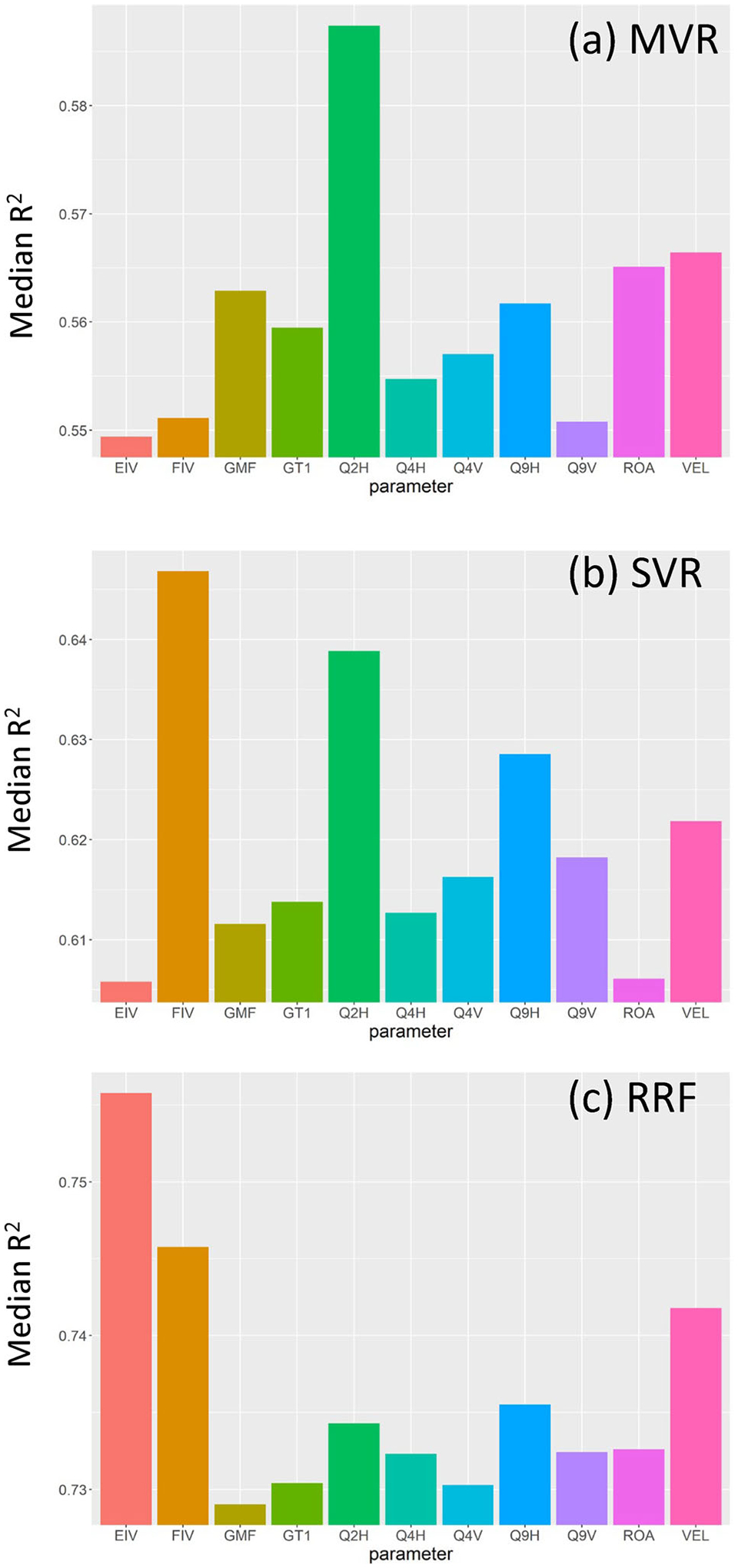
Relative importance of explanatory variables to models summarized as the median coefficient of variation from 1000-fold cross-validation for (a) multivariate linear regression (MVR), (b) support vector regression (SVR), and (c) random regression forest (RRF). See Table 1 for description of acronyms.

The relative variable importance for each ML model type, as evaluated through the 1000-fold cross-validation procedure and chosen based on the median *R*^2^ value produced from models associated with a given explanatory variable, are shown in Figure 11. From this evaluation, the three most important variables (in descending order) for each method were as follows:

1. MVR: Q2H, VEL, and ROA;
2. SVR: FIV, Q2H, and Q9H;
3. RRF: EIV, FIV, and VEL.

*R*^2^ for transferability tests using these parameters were of 0.11–0.37 (MVR), 0.61–0.67 (SVR), and 0.62–0.79 (RRF). RMSE values are also reported in Table 3.

These results reflect the ability of each individual parameter to influence model fits; however, to select an optimal set of explanatory variables for each ML method, we extracted the highest median *R*^2^ value across all cross-validation folds. The following best-fit parameter sets were selected through this analysis:

1. MVR: Q2H, VEL, and ROA (same as above);
2. SVM: Q2H, Q9H, and FIV (same as above);
3. RRF: Q2H, Q9H, FIV, and EIV.

Using these best-fit parameter sets, each ML method was again subjected to the transferability tests. Compared to using the full parameter set, the results from each method were generally better and more consistent between the two tests, achieving *R*^2^ values of 0.52–0.67 (MVR), 0.58–0.77 (SVR), and 0.73–0.79 (RRF). RMSE values for these tests are shown in Table 3.

**FIGURE 12:**
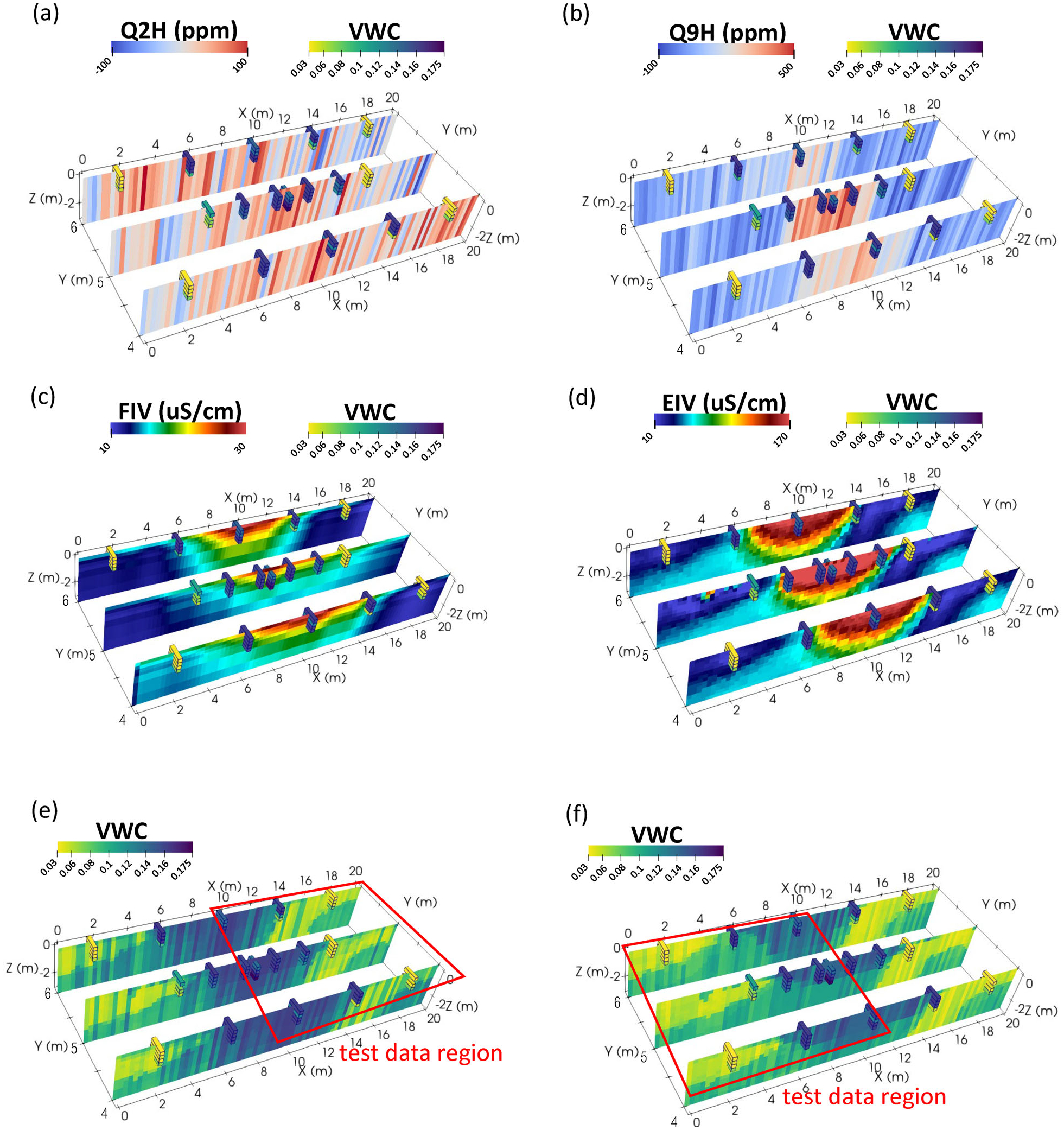
(a–d) Datasets from the irrigation experiment in Haddam Meadows, Connecticut, used in the final random regression forest (RRF) model compared with volumetric water content (VWC) values; (a) quadrature data at 19,110 Hz at horizontal coplanar orientation (Q2H); (b) quadrature data at 93,090 Hz at horizontal coplanar orientation (Q9H); (c) inverted, temperature-corrected FDEM electrical conductivity (FIV); (d) electrical conductivity output from post-irrigation inversion electrical resistivity tomography (EIV); (e–f) RRF model output from transferability test #1 and transferability test #2, respectively. Discrete soil moisture points are shown as outlined blocks.

Overall, RRF models consistently showed the highest *R*^2^ and lowest RMSE among the ML methods and outperformed petrophysically derived estimates of VWC (FEMVWC, GPRVWC, and ERTVWC), as can be seen in Table 2. Based on the transferability tests (Table 3), the best overall model (as judged by highest *R*^2^ and lowest RMSE) was an RRF model using Q2H, Q9H, FIV, and EIV. Plots of these explanatory variables, VWC, and the transferability test results are depicted in Figure 12.

## 4 | DISCUSSION

The overall best results were obtained using an RRF model that incorporates inverted ERT (EIV), inverted FDEM (FIV), and limited raw FDEM (Q2H and Q9H) data to predict VWC. Good performance was observed even when this model was used to predict VWC for a significant test portion (>50%) of the total dataset (Figure 12e,f). These testing data were physically separated from the training set and exhibited a wide range of moisture values (i.e., unsaturated to saturated).

The inverted ERT dataset (EIV) was ranked as most important to RRF models and was also included in the optimal RRF parameter set identified through cross-validation, but showed low importance in MVR and SVR models. VWC values derived from the EIV dataset through Archie’s Law also compared poorly to measured VWC. Though the spatial pattern of irrigation is captured in the EIV dataset (Figure 7c), the relation to VWC is nonlinear and not adequately modeled by an Archie relation (Figure 10a). The absolute magnitudes of inverted ERT data are heavily influenced by errors attributed to the raw ERT data, which are not sufficiently captured by stacking errors and are better assessed by collecting reciprocal datasets (e.g., Labrecque et al., 1996). In our case, the raw ERT errors were assumed based on experience and what was needed to achieve model convergence, that is, errors from out-of-irrigation electrodes had to be increased relative to electrodes within the irrigation area. Gathering reciprocal measurements was time-prohibitive given the transient conditions and an inability to capitalize on multiple instrument channels, which is particularly relevant for dipole–dipole data.

The complexities of accurate ERT inversion error modeling and petrophysical modeling are simplified for RRF as it does not need to fit an explicitly defined error model nor conform to a specified form of petrophysical model. The MVR and SVM methods have greater difficulty with nonlinearity, which is likely the reason the EIV dataset was not ranked as important for these methods. However, the raw apparent resistivity ERT data (ROA) ranked highly in importance for MVR and is not surprising given that the irrigated zone can be observed in the pseudosection data (Figure 7a), even if there are some noisy datapoints throughout. The relatively simple site conditions and common array geometries used in this study result in the conventional positioning of pseudosection data to be sufficiently spatially accurate, though this would likely break down under complex site conditions where significant distortion of the pseudosection is expected (Ritz et al., 1999). This indicates some potential for ML methods to potentially be used as a surrogate for geophysical inversion. For example, Aleardi et al. (2022) recently demonstrated an ML-based ERT inversion approach; however, they note that collecting a sufficiently large training dataset is challenging.

More advanced data collection and inversion procedures could potentially be used to improve ERT inversion results. Data were collected along a single 2D line and used a 3D inversion program to estimate out-of-plane EC, though sensitivity in the out-of-plane model elements to data is poor and could be improved with a truly 3D data collection scheme. Time-lapse inversion (e.g., J. Robinson et al., 2019) could also potentially be used to identify areas of changing VWC more accurately.

FDEM raw and inverted data (Q2H, Q9H, FIV) ranked highly in importance in both SVR and RRF models (Figure 11b,c) and were included in the optimal set of parameters for both methods. Compared to EIV, the FIV dataset showed a stronger linear correlation to VWC (Figures 8 and 10b), which likely explains its importance in SVM models. The inclusion of the raw Q2H and Q9H datasets may again indicate an ability of ML to bypass or alleviate some of the issues of geophysical inversion (i.e., smoothing). Despite a lack of specific depth information, these datasets have different sampling volumes (i.e., Q9H = smaller/shallow, Q2H = larger/deeper), which perhaps effectively serve to sharpen the spatially smoothed EC estimates from the FIV dataset in SVR and RRF models. However, it is important to note that the importance of FDEM data to prediction of VWC in this study likely depends on the elevated specific conductance of the irrigation water used. Pre-modeling (see Supplemental Material) indicated that a 1-m thick layer containing pore water with a specific conductance of 1000 μS/cm would generate sufficient signal to be observable in the upper three HCP and VCP quadrature frequencies we used, which reflected what was observed (Figure 6). Further, FDEM data can be highly sensitive to near-surface temperature variation. For example, we performed some simple modeling to show that a drop from 30 to 25°C in a 100 Ωm soil is enough to affect an apparent −229 ppm change to 93,090 Hz HCP data, which is approximately the drift correction value applied to this frequency and sensor orientation at our site. Though this is not ideal, it does indicate another benefit to ML models to bypass issues with instrument calibration (Loose et al., 2020).

Extraneous datasets tend to detract from the quality of predictions from ML methods (Gupta & Gupta, 2019) similar to those used in this study; behavior that was shown in our models when evaluated by cross-validation and tested in a robust way (Table 3). Training ML models on data that are not sensitive to the parameter of interest (VWC) has an adverse effect as it forces algorithms such as RRF to try to develop (potentially meaningless) relationships within these models. Recursive variable elimination, whereby the least important explanatory variables are removed in a stepwise manner, is one method for handling this issue. In this study, it was unexpected that including GPR velocity information in the RRF model did not lead to improvement, given that these data have strong linear correlations to VWC (Figure 8). GPR velocity (VEL) scored as the fourth most important explanatory variable in the RRF cross-validation tests (Figure 11c), yet the RRF model was ultimately able to make better and more transferrable predictions without including this information.

It is possible that additional observational data would improve the ability of ML models to incorporate other explanatory variables clearly sensitive to soil moisture (e.g., VEL). The dataset used to train ML models in this study had a limited number (66) of reference VWC observations, of which only 40 observations were used to train models (with a maximum of 17 explanatory variables) in the cross-validation procedure. This is smaller than what is typically recommended for regression problems. For example, Harrel (2015) suggests at least 10–20 observations per explanatory variable.

We recognize that the model developed for this work is likely specific to this site and experiment. While ERT was able to identify the conductive irrigation bulb, the natural resistive groundwater at the test site was largely undetectable. Similarly, the efficacy of FDEM was limited by naturally resistive conditions due to insufficient signal/noise. At sites with variable soil salinity, or variable EC due to soil materials (e.g., clay versus sand) VWC-EC relations may be complicated and would require a calibration training/test set that fully captures the range of subsurface conditions. Additional geophysical methods could be particularly useful at more complex sites, like the addition of common midpoint surveys from GPR which could significantly improve the velocity (and therefore moisture) characterization. Nevertheless, we have demonstrated here the efficiency of ML methods for developing site-specific relationships by capitalizing on the data “that are” as opposed to “what could be” given infinite resources and expertise.

## 5 | CONCLUSIONS

Geophysical results from surface-deployed systems commonly compare poorly in terms of point-to-point correlation with directly measured moisture data. This is due to the many uncertainties and sometimes subjective choices involved in bringing raw geophysical data to a final moisture estimate, spatial smoothing introduced and commonly necessary for stable geophysical inversions, and inherent differences in measurement volume between geophysical data and that from moisture sensors. Also, the directly measured moisture data itself may have large (and difficult to quantify) uncertainties due to disturbance from probe installation and the specific technique used. Further, combining data types for moisture estimation can be limited to qualitative comparisons of results given uncertainty in the petrophysical relation between geophysical properties and moisture. Although advanced inversion codes and/or geostatistical methods can fill this need, they may yet remain overly restrictive in terms of physical and/or statistical assumptions for many sites lacking detailed characterization. ML methods offer a viable solution to bypass complicated, and potentially subjective and uncertain, approaches to data fusion and parameter estimation.

Despite the benefits of ML approaches, these methods have significant limitations. Common algorithms require data to be made available in a table format, necessarily requiring a user to link the parameter of interest with a specific data value for every input dataset, which can be challenging for soil moisture estimation given that each measurement may not be collocated, nor associated with a specific depth. Further, missing values must be accounted for. For multi-method datasets, this introduces subjective choices in terms of how to interpolate/extrapolate data into areas covered by one method but not by another.

ML methods are conceived to be as accurate as possible with a provided dataset; therefore, it is crucial to validate ML model efficacy with meaningful testing to avoid overfitting. Here, we performed an experiment where we evaluated three ML algorithms (multivariate linear regression, support vector regression, and random regression forests) on all combinations of available explanatory variables (FDEM raw and inverted data, ERT raw and inverted data, and GPR raw and interpreted data). For each of these evaluations, we additionally performed 1000-fold cross-validation, shuffling our 66 direct moisture observations and associated geophysical datasets in 39–27 training-test splits over 1000 folds. From these tests, we were able to approximately assess the relative importance of each explanatory variable to VWC predictions, and to select an optimal model based on the highest median prediction accuracy from all tests. In this case, the best model used a random regression forest and inverted ERT, FDEM, and 2/5 frequencies of the horizontal coplanar FDEM data, though other variables ranked highly in importance (such as interpreted GPR velocities). We further tested the intra-site transferability of this model in a more rigorous evaluation that physically divided the site into two halves (one half used for training and the other half used for testing).

We recognize the selected model from this study is site specific, and possibly experiment specific; however, the overall approach could be used to develop geophysical moisture relations at other sites. We do not intend to suggest that ML methods could or should replace physics-driven models; however, they can be useful tools to augment these models or bypass potentially problematic assumptions for geophysical inversions or petrophysical models.

## Supplementary Material

Supplement1

## Figures and Tables

**TABLE 1:** Acronyms, geophysical method, processing level (raw versus processed), and descriptions for variables used in this paper

Acronym	Method	Unit	Processing level	Description
VWC	Moisture probe (direct measurement)	Water content (dimensionless proportion)	Raw	Volumetric water content determined from soil moisture sensors
Q0H	Frequency domain electromagnetics	Parts per million	Raw	Quadrature data at 3930 Hz at horizontal coplanar orientation
Q1H	Frequency domain electromagnetics	Parts per million	Raw	Quadrature data at 8670 Hz at horizontal coplanar orientation
Q2H	Frequency domain electromagnetics	Parts per million	Raw	Quadrature data at 19,110 Hz at horizontal coplanar orientation
Q4H	Frequency domain electromagnetics	Parts per million	Raw	Quadrature data at 42,210 Hz at horizontal coplanar orientation
Q9H	Frequency domain electromagnetics	Parts per million	Raw	Quadrature data at 93,090 Hz at horizontal coplanar orientation
Q0V	Frequency domain electromagnetics	Parts per million	Raw	Quadrature data at 3930 Hz at vertical coplanar orientation
Q1V	Frequency domain electromagnetics	Parts per million	Raw	Quadrature data at 8670 Hz at vertical coplanar orientation
Q2V	Frequency domain electromagnetics	Parts per million	Raw	Quadrature data at 19,110 at vertical coplanar orientation
Q4V	Frequency domain electromagnetics	Parts per million	Raw	Quadrature data at 42,210 at vertical coplanar orientation
Q9V	Frequency domain electromagnetics	Parts per million	Raw	Quadrature data at 93,090 at vertical coplanar orientation
ROA	Electrical resistivity tomography	Ohm-meters	Raw	Raw apparent electrical resistivity from ERT pseudosection
LAA	Ground penetrating radar	Millivolts	Raw	Vertical sum of logarithm of absolute GPR amplitudes
GMF	Ground penetrating radar	Megahertz	Raw	Mean of the power spectrum from fast Fourier transform of GPR amplitudes
GT1	Ground penetrating radar	Nanoseconds	Raw/processed	Picked first arrival time to upper reflector in GPR data
EIV	Electrical resistivity tomography	Microsiemens Per centimeter	Processed	Electrical conductivity from 2D L2-norm geophysical inversion (R3t)
FIV	Frequency domain electromagnetics	Microsiemens per centimeter	Processed	Electrical conductivity through 1D laterally constrained inversion (Workbench)
VEL	Ground penetrating radar	Meters per nanosecond	Processed	Radar electromagnetic velocity from diffraction hyperbola and two-way travel time to interface picking
FEMVWC	Frequency domain electromagnetics	Water content (dimensionless proportion)	Processed and petrophysical model applied	Volumetric water content estimated from inverted FDEM data using Archie’s Law
ERTVWC	Electrical resistivity tomography	Water content (dimensionless proportion)	Processed and petrophysical model applied	Volumetric water content estimated from inverted ERT data using Archie’s Law
GPRVWC	Ground penetrating radar	Water content (dimensionless proportion)	Processed and petrophysical model applied	Volumetric water content estimated from processed GPR data using Topp’s empirical equation

Abbreviations: 1D, one-dimensional; ERT, electrical resistivity tomography; FDEM, frequency domain electromagnetics; GPR, ground penetrating radar; GMF, GPR mean frequency; LAA, log absolute amplitude; VWC, volumetric water content.

**TABLE 2:** Comparison of volumetric water content (VWC) predictions from various methods. See Table 1 for description of acronyms

VWC model	All-in *R*^2^ (RMSE)	Cross-validation median *R*^2^ (range)	Transferability test #1 *R*^2^ (RMSE)	Transferability test #2 *R*^2^ (RMSE)
FEMVWC	0.35 (0.043)	NA	NA	NA
GPRVWC	0.49 (0.045)	NA	NA	NA
ERTVWC	0.16 (0.970)	NA	NA	NA
MoisturEC	1 (0)	0.79 (0.46–0.91)	0.63 (0.037)	0.41 (0.053)
MVR	0.71 (0.028)	0.64 (0.20–0.86)	0.67 (0.037)	0.52 (0.039)
SVR	0.76 (0.026)	0.75 (0.18–0.92)	0.77 (0.029)	0.58 (0.040)
RRF	0.94 (0.013)	0.79 (0.48–0.94)	0.79 (0.026)	0.73 (0.036)

Abbreviations: ERT, electrical resistivity tomography; GPR, ground penetrating radar; MVR, multivariate linear regression; NA, not applicable; RMSE, root-mean-square error; RRR, random regression forests; SVR, support vector regression; VWC, volumetric water content.

**TABLE 3:** Transferability test results for machine learning (ML) models using different parameter sets. See Table 1 for description of acronyms

VWC model	All-in *R*^2^ (RMSE)	Three most important parameters *R*^2^ (RMSE)	Optimal parameter set *R*^2^ (RMSE)
MVR	0.11–0.37 (0.077–0.087)	Q2H, VEL, ROA 0.52–0.67 (0.037–0.039)	Q2H, VEL, ROA 0.52–0.67 (0.037–0.039)
SVR	0.61–0.67 (0.034–0.040)	FIV, Q2H, Q9H 0.58–0.77 (0.029–0.040)	FIV, Q2H, Q9H 0.58–0.77 (0.029–0.040)
RRF	0.53–0.79 (0.028–0.042)	EIV, FIV, VEL 0.62–0.79 (0.026–0.038)	Q2H, Q9H, FIV, EIV 0.73–0.79 (0.026–0.036)

Abbreviations: MVR, multivariate linear regression; RRR, random regression forests; SVR, support vector regression.

## Data Availability

Data from the irrigation experiment are available in a USGS public data release (Terry et al., 2022).

## Core Ideas

- Various geophysical methods (e.g., frequency domain electromagnetics [FDEM], ground penetrating radar, electrical resistivity tomography [ERT]) are sensitive to soil moisture (volumetric water content [VWC]).
- Machine learning provides methods for data fusion and less need for assumptions/advanced data processing.
- Raw and processed geophysical data were evaluated in traditional and machine learning models to predict VWC.
- Random regression forest models using FDEM and ERT information gave the highest overall VWC accuracy.
- Models yielded good results even when trained on only half of a physically separated portion of the dataset.

## APPENDIX: This appendix provides additional details of the ML methods used in this paper

### Multivariate regression

Multivariate regression seeks to estimate model coefficients (**w**) for all explanatory variables (**x**) by minimizing the squared error between observed values (**y**) and predictions from the model,

(A1)
$$\text{min}\overset{n}{\underset{i=1}{\sum }}{\left(y_{i}-w_{i}x_{i}\right)}^{2}$$

where *n* is the number of observations. This optimization problem can be quickly solved with linear algebra.

### Support vector regression

Unlike MVR which optimizes the model coefficient vector (**w**) based on the best fit to observed data points, the goal of support vector regression is instead to minimize the L2-norm of **w** subject to a maximum error constraint, *ε*. By minimizing the L2-norm of **w**, the algorithm seeks a solution that is as “flat” as possible, while *ε* allows a certain amount of flexibility in the optimized solution. Nevertheless, outliers in the data may have large errors that are outside of the desired *ε*. That is why SVR includes the so-called “slack variables”, ξ, which allow for deviation for any point from the maximum error constraint:

(A2)
$$\text{min}\frac{1}{2}w^{2}+C\overset{n}{\underset{i=1}{\sum }}\xi_{i}$$

subject to

(A3)
$$\left|y_{i}-w_{i}x_{i}\right|\leq \varepsilon +\left|\xi_{i}\right|$$

where *C* is the “box constraint”: a tunable regularization parameter with greater values allowing for larger model tolerance.

To solve the above minimization problem, Lagrange multipliers are commonly used. This method allows for a nonlinear mapping of observations to high order space and is possible through a kernel function, such as a Gaussian Radial Basis Function.

### Random regression forests

The RRF algorithm is built on regression trees (most commonly, the CART algorithm). Regression trees are decision trees that are applied in a regression context (i.e., the output is a continuous variable as opposed to a categorical variable). Starting with an initial binary split based on one of the explanatory variables, the tree is formed by successive binary splits to explanatory variables. In other words, the tree consists of an ordered set of conditional rules that end with a prediction for the value of the independent variable given those sets of conditions. Development of the trees uses a numerical approach whereby various splits are tested and are chosen based on minimizing the squared error between observed values and predictions from the model.

Though this effectively allows for a nonlinear mapping between explanatory variables and the independent variable, it is easy to see that a tree with many splits could be overoptimized to the point of perfect fits to training data, that is, there is a long series of rules to predict every individual data point. To somewhat limit overfitting, additional constraints on the maximum level of splits (maxdepth) and minimum number of observations each split can contain (minsplit) are used, however even this approach can yield quite unstable models for complicated real-world applications.

The RRF method seeks to overcome this issue by developing an ensemble prediction based on many decision trees, each of which only uses a subset of the explanatory variables. Several (n_tree) regression trees are formed using a subset (m_try) of the explanatory variables, and the final predictions are calculated as the average prediction from all the individual decision trees.

## Abbreviations:

1D: one-dimensional
2D: two-dimensional
3D: three-dimensional
ABS: log absolute amplitude
AGC: automatic gain control
DOI: depth of investigation
EC: electrical conductivity
EM: electromagnetic
ERT: electrical resistivity tomography
ERTVWC: ERT-derived volumetric water content
FDEM: frequency domain electromagnetics
FEMVWC: FDEM-derived volumetric water content
GPR: ground penetrating radar
GPRVWC: GPR-derived volumetric water content
GPS: global positioning system
HCP: horizontal coplanar
LAA: summed log absolute amplitude
ML: machine learning
MVR: multivariate regression
NMR: nuclear magnetic resonance
RMSE: root-mean-squared error
RRF: random regression forests
SVR: support vector regression
TDR: time-domain reflectometer
VCP: vertical coplanar
VWC: volumetric water content (soil moisture).
