# Supplementary material for: Field evaluation of semi-automated moisture estimation from geophysics using machine learning
Source: Vadose Zone J. Author manuscript; Available in PMC 2024 Mar 1. (PMC10494895; doi:10.1002/vzj2.20246)
Supplement: Supplement1 [file NIHMS1919740-supplement-Supplement1.docx]

Supplementary material

We performed simple pre-modeling of electrical geophysical data prior to our irrigation experiment. These modeling efforts are described below.

Electrical resistivity tomography (ERT)

The feasibility of ERT surveys for irrigation monitoring were evaluated with SEER (Terry et al., 2017). As a rough approximation, we assumed an Archie relation where the specific conductance of the water was 1,000 μS/cm (10 ohmm), interconnected porosity was 0.15, the saturation exponent was 1, and the cementation exponent was 2. This equates to an approximate bulk soil resistivity of 444 ohmm. We modeled 0.5-meter electrode spacing, 5% errors, and a combined Wenner and dipole-dipole survey geometry. Results are shown in Figure S1 and indicated good ability to delineate the moisture bulb under these conditions.

Frequency domain electromagnetics (FDEM)

Relative to ERT, fresh groundwater is potentially difficult to detect with FDEM without a strong electrical conductivity contrast with the soil. We performed simple 1D FDEM forward modeling using a similar resistivity model as above (3-m thick 444 ohmm layer over 1,000 ohmm layer). An R dashboard app for interactively evaluating FDEM forward models was used to simulate this situation and indicated that frequencies above approximately 10,000 Hz will be sufficiently sensitive to detect the moisture anomaly, given typical ambient noise levels (75 ppm) observed in a wide range of field conditions with a GEM-2 instrument across all frequencies.

This pre-modeling further indicates that precise changes in soil moisture may be fundamentally difficult to detect. For example, we also modeled a change in the thickness of the irrigated zone from 1 meter to 3 meters thick. Here only the higher frequencies (> 50,000 Hz) showed sufficient sensitivity (e.g., a change in data values > 75 ppm) in response to this change.

| 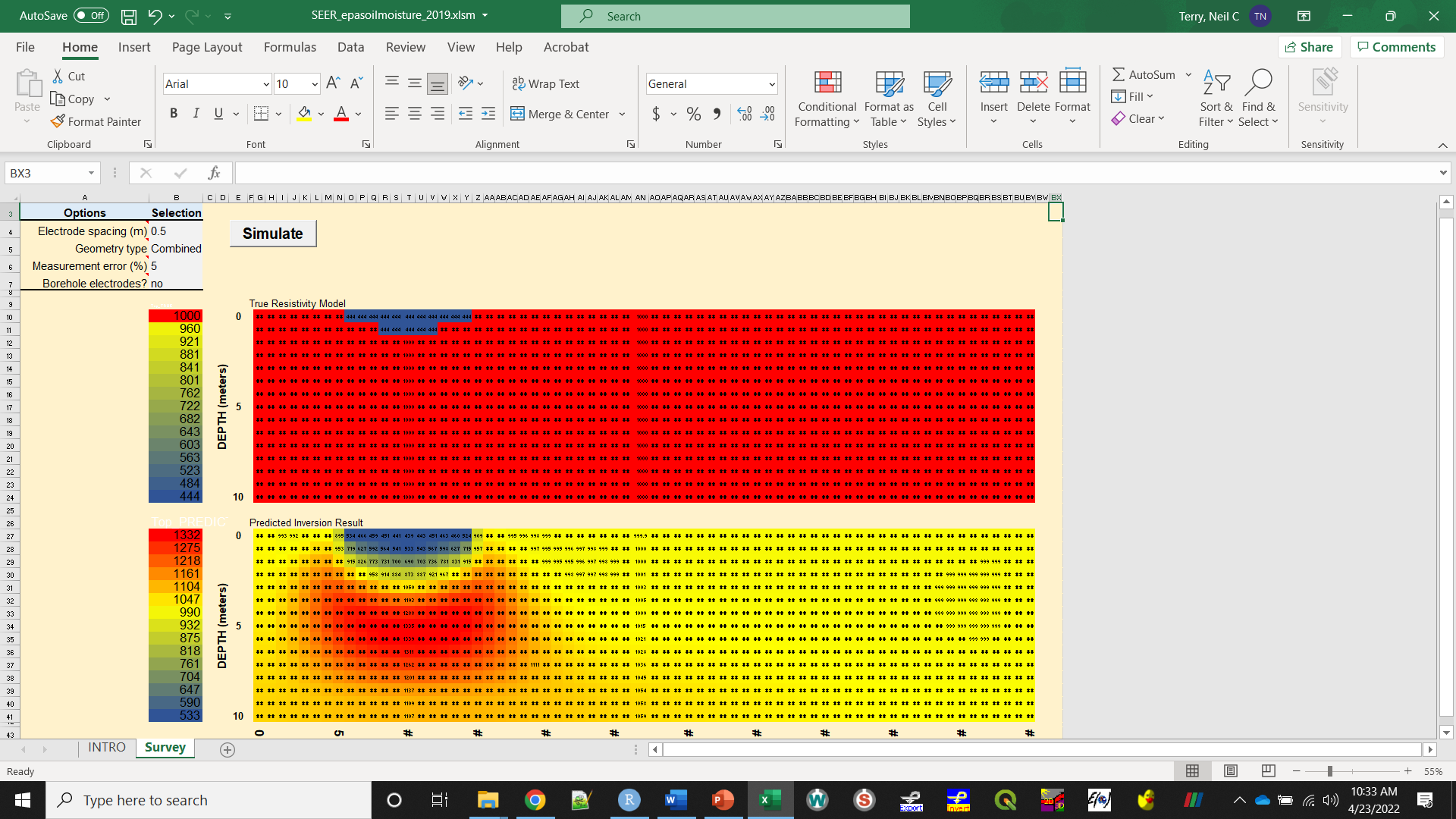 |
| --- |
| **Figure S1.** Screenshot of SEER resistivity pre-modeling tool to evaluate resistivity surveys for the irrigation experiment in this study. |

| 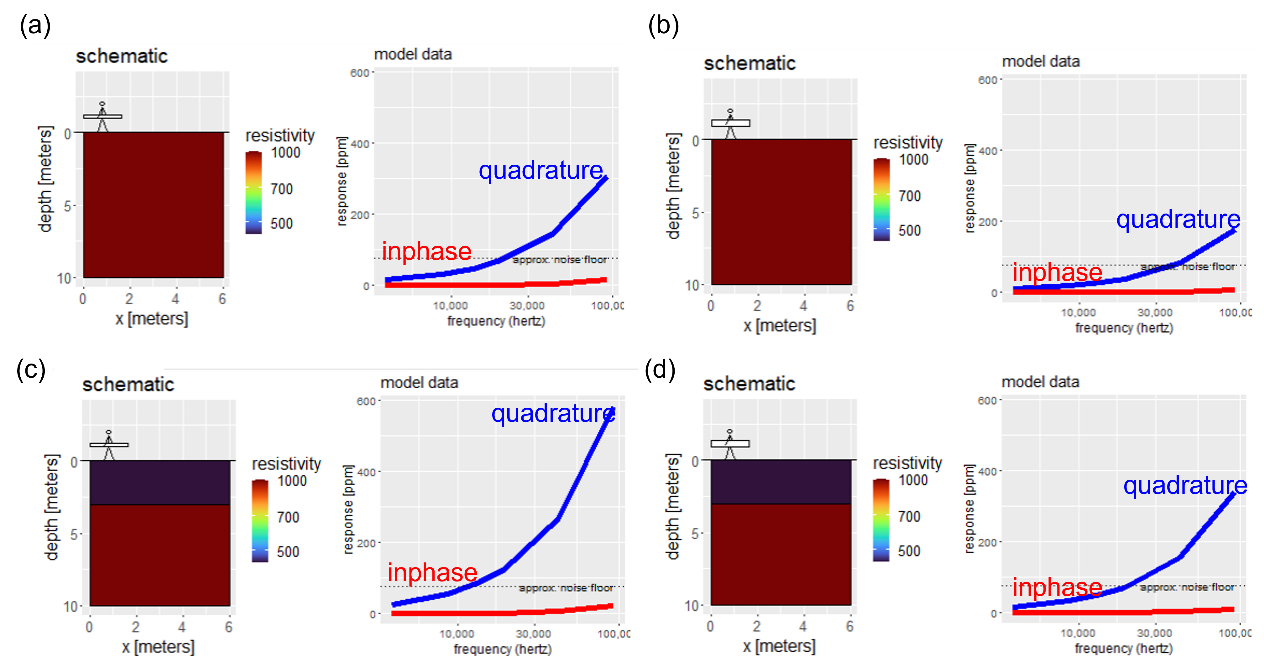 |
| --- |
| **Figure S2.** Screenshots of frequency domain electromagnetics (FDEM) pre-modeling data used to evaluate likely data sensitivity to the irrigation experiment in this study. Screenshots show response to changes in model and coil orientation; (a) horizontal coplanar (HCP) coil orientation and 1,000 ohmm (dry) soil; (b) vertical coplanar (VCP) coil orientation and 1,000 ohmm (dry) soil; (c) HCP coil orientation with 444 ohmm (wet) zone; (d) VCP coil orientation with 444 ohmm (wet) zone. |

**Disclaimer:** Use of trade, firm, or product names is for descriptive purposes only and does not imply endorsement by the U.S. Government.

**References**

Terry, N., Day-Lewis, F.D., Robinson, J.L., Slater, L.D., Halford, K., Binley, A., Lane, J.W., Jr., & Werkema, D. (2017). Scenario Evaluator for Electrical Resistivity Survey Pre-Modeling Tool. *Groundwater*, 55, 885–890. doi: <http://dx.doi.org/10.1111/gwat.12522>.
